# Sustained Radiosensitization of Hypoxic Glioma Cells after Oxygen Pretreatment in an Animal Model of Glioblastoma and *In Vitro* Models of Tumor Hypoxia

**DOI:** 10.1371/journal.pone.0111199

**Published:** 2014-10-28

**Authors:** Ryon H. Clarke, Shayan Moosa, Matthew Anzivino, Yi Wang, Desiree Hunt Floyd, Benjamin W. Purow, Kevin S. Lee

**Affiliations:** 1 Department of Neuroscience, University of Virginia, Charlottesville, VA, United States of America; 2 School of Medicine, University of Virginia Health System, Charlottesville, VA, United States of America; 3 Division of Neuro-Oncology, Departments of Neurology, Microbiology, and Biochemistry and Molecular Genetics, University of Virginia Health System, Charlottesville, VA, United States of America; 4 Department of Neurological Surgery, University of Virginia, Charlottesville, VA, United States of America; University of Oxford, United Kingdom

## Abstract

Glioblastoma multiforme (GBM) is the most common and lethal form of brain cancer and these tumors are highly resistant to chemo- and radiotherapy. Radioresistance is thought to result from a paucity of molecular oxygen in hypoxic tumor regions, resulting in reduced DNA damage and enhanced cellular defense mechanisms. Efforts to counteract tumor hypoxia during radiotherapy are limited by an attendant increase in the sensitivity of healthy brain tissue to radiation. However, the presence of heightened levels of molecular oxygen during radiotherapy, while conventionally deemed critical for adjuvant oxygen therapy to sensitize hypoxic tumor tissue, might not actually be necessary. We evaluated the concept that pre-treating tumor tissue by transiently elevating tissue oxygenation prior to radiation exposure could increase the efficacy of radiotherapy, even when radiotherapy is administered after the return of tumor tissue oxygen to hypoxic baseline levels. Using nude mice bearing intracranial U87-luciferase xenografts, and *in vitro* models of tumor hypoxia, the efficacy of oxygen pretreatment for producing radiosensitization was tested. Oxygen-induced radiosensitization of tumor tissue was observed in GBM xenografts, as seen by suppression of tumor growth and increased survival. Additionally, rodent and human glioma cells, and human glioma stem cells, exhibited prolonged enhanced vulnerability to radiation after oxygen pretreatment *in vitro*, even when radiation was delivered under hypoxic conditions. Over-expression of HIF-1α reduced this radiosensitization, indicating that this effect is mediated, in part, via a change in HIF-1-dependent mechanisms. Importantly, an identical duration of transient hyperoxic exposure does not sensitize normal human astrocytes to radiation *in vitro*. Taken together, these results indicate that briefly pre-treating tumors with elevated levels of oxygen prior to radiotherapy may represent a means for selectively targeting radiation-resistant hypoxic cancer cells, and could serve as a safe and effective adjuvant to radiation therapy for patients with GBM.

## Introduction

Primary malignant gliomas are the most common type of brain cancer in adults, with an estimated 23,000 people newly diagnosed each year in the U.S. [1]. Glioblastoma multiforme (GBM) - the most common and deadly form of these tumors - remains an incurable disease, and even with the most aggressive treatment protocols available the average life expectancy for patients diagnosed with GBM is 12–15 months [2]. The World Health Organization (WHO) classifies GBM as a Grade IV primary brain tumor, characterized by its rapid cell proliferation, cellular atypia, angiogenesis, and aggressive invasion of tumor cells into healthy brain tissue [3]. The GBM tumor mass also displays areas of ischemia and necrosis. The rapid rate of tumor growth can outstrip neovascularization, creating diffusion distances too great to provide sufficient blood flow, and poorly orchestrated angiogenesis can lead to incompetent, leaky, and blunted blood vessels [4]–[6]. Tumor hypoxia in GBM is an important factor in tumor aggression and progression, but also represents a significant impediment for the success of chemo- and radiotherapies [7], [8].

Resistance to radiotherapy in ischemic regions of tumors is generally attributed to reduced concentrations of dissolved molecular oxygen in the tissue and a subsequent reduction in the production of reactive oxygen species (ROS) during radiation exposure. ROS directly damage multiple cellular elements and are critical for the inhibition of DNA repair [9]–[11]. Additionally, many cellular and molecular changes occur in response to decreased oxygen levels, mediated largely by the transcription factor hypoxia inducible factor-1 (HIF-1) [12]. Among the hundreds of gene targets for HIF-1 are those that regulate cellular metabolism, angiogenesis, apoptosis, cell motility, growth and proliferation, all of which may provide cancer cells with a treatment-resistant and aggressive phenotype during periods of hypoxic stress [4], [13]–[17].

The well-established link between tumor hypoxia, HIF-1 expression and tumor resistance to radiation therapy has prompted a number of experimental and clinical efforts to either counteract or exploit hypoxic regions in fast growing tumors such as GBM. Included in these efforts are normobaric and hyperbaric oxygen treatments, oxygen mimetics, hypoxic cell radiosensitizers, and vascular normalization [18]–[23]. However, a serious drawback to many of these experimental approaches lies in the non-selective nature of the treatments. While these strategies can increase the vulnerability of glioma tissue to radiation, they can also radiosensitize healthy peritumoral brain tissue, creating a higher risk to healthy, non-target cells. Although modern clinical imaging and radiotherapeutic approaches continue to evolve, allowing for a more targeted delivery of radiation to the bulk of the tumor volume in GBM [24]–[26], the highly invasive nature of glioma cells means that damage to peritumoral brain tissue during treatment remains a valid concern. It is therefore important to develop methods of selectively sensitizing glioma cells during radiation therapy in order to better slow tumor progression and limit collateral damage to healthy brain tissue.

In the present study, we investigated an approach by which hypoxic glioma cells and human glioma stem cells (GSCs) can be sensitized to ionizing radiation following transient exposure to normoxic conditions. Using nude mice bearing orthotopic U87-luciferase xenografts, and *in vitro* models of tumor hypoxia, our findings indicate that briefly exposing hypoxic cancer cells to normoxic or near-normoxic levels of oxygen, and then returning these cells to baseline hypoxic conditions, provides a window of vulnerability to ionizing radiation in the absence of elevated levels of molecular oxygen. This enhanced glioma cell vulnerability is mediated, at least in part, by a change in the activity of HIF-1. Notably, exposing normoxic human astrocytes to transient hyperoxia *in vitro* before radiation exposure does not sensitize these cells to treatment, further indicating the selective vulnerability of hypoxic tumor cells afforded by this type of oxygen therapy.

## Methods

### Ethics statement

Primary human GBM cultures enriched for glioma stem cells (GSCs) termed 0308 were initially obtained from surgical resections from GBM patients (“Following informed consent, human tumor samples classified as GBM based on World Health Organization (WHO) criteria were obtained from patients undergoing surgical treatment at the National Institutes of Health in accordance with the appropriate Institutional Review Boards.”) as previously described [27]. The University of Virginia Institutional Animal Care and Use Committee (IACUC) approved all procedures involving animals in this study (protocol #3819). All surgeries were carried out under ketamine/dexmedetomidine, and all efforts were made to minimize suffering.

### Cell culture

The U87 cell line (American Type Culture Collection) and a U87 cell line genetically engineered to expresses the firefly luciferase gene (U87-luc) (a generous gift from Hong Zhong at Parabon Nanolabs) were maintained in Dulbecco’s minimal essential medium (Life Technologies) supplemented with 10% fetal bovine serum (FBS) and 1% penicillin-streptomycin. The GL261 cell line (NCI, Frederick Repositories) was maintained in RPMI medium (Life Technologies) supplemented with 10% FBS and 1% penicillin-streptomycin. The previously validated 0308 glioma stem cell line [27] was obtained from Dr. Jeongwu Lee and was maintained in non-treated culture flasks in Neurobasal media (Life Technologies) supplemented with 1% B27, 0.5% N2, 0.01% FGF, 0.1% EGF, 0.3% L-Glutamine, and 1% penicillin-streptomycin. The normal human astrocyte cell line (National Institutes of Health) was maintained in F12/MEM (Life Technologies) supplemented with 10% FBS and 1% penicillin-streptomycin. All cell lines were maintained at 37°C in a humidified incubator containing 5% CO_2_ and 21% O_2_.

### Human tumor xenografts in nude mice

All experiments with animals were performed in accordance with protocols approved by the University of Virginia Institutional Animal Care and Use Committee (IACUC). 14–16 week old nude female mice (National Cancer Institute) were anesthetized with ketamine (50 mg/kg) and dexmedetomidine (0.5 mg/kg) administered i.p. and placed in a stereotactic frame. A midline scalp incision was made and a burr hole was drilled in the right-side of the skull 2.3 mm lateral to the midline and 0.1 mm posterior to the bregma. A 10 µl Hamilton syringe, containing 250,000 U87-luc cells in 4 µl, was positioned in the brain at a depth of 2.35 mm from the cortical surface and cells were injected into the striatum at a rate of 0.3 µl/min. After cell injection, the syringe was slowly removed, the burr hole was sealed with bone wax, and the scalp was sutured together. Post-operatively, animals were administered s.c. buprenorphine (0.1 mg/kg) and antisedan. Tumors were allowed to grow for 14 days, at which time imaging and treatments were initiated.

The nude-U87 glioma model is a well-established, well-characterized orthotopic xenograft model [28]–[30]. The benefits of this model include the fact that the U87 cell line is a well-defined, widely-recognized human glioma cell line that exhibits tumor characteristics in nude mouse glioma models that mimic glioma cell behavior in patient tumors, particularly those pertaining to the tumor vasculature [30]. While this tumor model displays less infiltrative growth properties and greater tumor encapsulation than certain other established glioma cell lines, the more predictable tumor vascularity that accompanies the U87 model was well suited for the current studies.

### Tissue oxygen measurements

Tumor-bearing animals (n = 3) at day 14 post-tumor implant (PTI) were sedated using a mixture of ketamine (30 mg/kg) and dexmedetomidine (0.3 mg/kg) and placed in a stereotactic frame. For tumor tissue monitoring, the same burr hole drilled for tumor implantation was used to position a Licox oxygen probe (Integra Neurosciences) at a depth of 2.35 mm from the cortical surface - the same coordinates at which glioma cells were injected. For monitoring of healthy striatal brain tissue in the same animal, a new burr hole was drilled in the contralateral skull 2.3 mm lateral to the midline and 0.1 mm posterior to the bregma, and the oxygen probe was positioned at a depth of 2.35 mm from the cortical surface. The same oxygen probe was used in a given animal for monitoring the contralateral brain and the tumor. Tissue pO_2_ (tpO_2_) readings were taken every minute for 20 minutes under ambient air (21% O_2_) respiration in order to establish a baseline. Fraction of inspired oxygen (FiO_2_) was then increased to 100% O_2_ for 25 minutes, after which it was returned to 21% O_2_ for the remainder of the observation period. All recorded tpO_2_ values were converted to a percentage of the baseline value obtained from the normal neural tissue in the contralateral brain. Animals used for tissue oxygen monitoring were not included in the subsequent survival study.

### Hyperoxic treatment and *in vivo* irradiation

Based on *in vivo* tumor measurements (described below), animals implanted with U87-luc cells were assigned to experimental groups on day 14 PTI in order to achieve an equivalent distribution of tumor sizes among groups. On the day of radiation treatment (day 14 PTI), tumor-bearing animals were sedated using a mixture of ketamine (30 mg/kg) and dexmedetomidine (0.3 mg/kg). Animals receiving 100% FiO_2_ pretreatment were placed in a modified gas mask apparatus that covered the entire snout of the animal and administered 100% oxygen for 25 minutes. Oxygen-treated animals were administered a whole-head dose of 8 Gy radiation at a rate of 3.3 Gy/min starting 25 minutes after the cessation of hyperoxic pretreatment. The 8 Gy dose selected for the *in vivo* experiments represents a slightly lower dosage compared with similar work using radiation treatment in the same animal model [31], [32]. We selected a dosage in the lower range in order to assess the postulated enhanced sensitivity to radiation produced by oxygen pretreatment. Animals that did not receive hyperoxic pretreatment instead breathed ambient air, and the same sedation and irradiation procedures were used. Radiation was administered using a SARRP research platform (Xstrahl Life Sciences) with a lead body shield. The *in vivo* radiation treatment study was done in two stages. Stage 1 was a preliminary study of 8 animals per group. Data from stage 1 were then used to estimate the number of animals needed to have 80% power under different assumptions about effect sizes consistent with the stage 1 data.

### *In vivo* tumor measurements

Orthotopic U87-luc tumors were visualized using an In Vivo Imaging System (IVIS; Xenogen Corp.) at day 14, 18, 22, 27, 31, 36, 41, and 46 PTI, or up until day of death. Twelve minutes prior to imaging, mice were given an i.p. injection of D-luciferin (Gold Biotechnology) (150 mg/kg). Five minutes prior to imaging, mice were initially anesthetized by placement in a chamber containing 5% Isofluorane, and anesthesia was maintained at 1–1.5% Isofluorane inside the IVIS to obviate movement during imaging. The light emitted from the luciferase-expressing U87 tumor cells of the nude mice was detected with the IVIS camera. Quantification and normalization of light intensity and photon counts were performed using Living Image software (Xenogen Corp.).

### Determination of Humane Endpoints

Tumor-bearing mice were housed in a barrier vivarium facility and evaluated twice daily for signs of deteriorating physical and behavioral health with the assistance of veterinary technicians. Humane endpoints were determined according to a clinical scoring system based on that outlined in the University of Virginia Guidelines and Policy for Determination of Humane Endpoints for Vertebrate Animals Used in Research. Animals were checked for their health during their entire survival period using a scoring scale consisting of 4 categories: Body Weight Changes, General Activity, Eyes and Nose Discharge, and Posture. Total scores of 7 or greater, or maximum scores in at least 2 categories warranted immediate euthanasia. Any animal with a weight loss of 20% or more was euthanized. Individual records were maintained for the life span of each animal. When criteria were met for euthanasia, mice were anesthetized with ketamine (50 mg/kg) and dexmedetomidine (0.5 mg/kg) and perfused intracardially, first with PBS and then with a 4% paraformaldehyde solution. Brains were saved for further analysis.

### *In vitro* hypoxic/hyperoxic exposure and radiation treatment

Cells were subjected to varying levels of oxygen in a Galaxy 14S incubator with oxygen control (New Brunswick Scientific). For hypoxic conditions, the chamber was charged with N_2_ and CO_2_ to produce gas concentrations of 1–5% O_2_ and 5% CO_2_ at 37°C during graded chronic hypoxia (GCH) or rapid acute hypoxia (RAH). The GCH and RAH protocols are described in greater detail in the Results section. For transient reoxygenation, cells were transferred to another incubator containing 5% CO_2_ and 21% O_2_ for 25 minutes at 37°C. Return to 1% O_2_ for radiation exposure was achieved by placing cells in a modular incubator chamber (Billups-Rothenberg) and flushing with a 95% N_2_/5% CO_2_ gas mixture for 4 minutes at 5 psi. For hyperoxic conditions, cells were placed in a Galaxy 14S incubator and the chamber was charged with a gas mixture containing 5% CO_2_ and 95% O_2_ until oxygen levels within the incubator reached 50%. Cells were maintained at this oxygen level for 25 minutes and then returned to an incubator containing 5% CO_2_ and 21% O_2_ for 25 minutes at 37°C prior to radiation. For radiation treatment, all cells were γ-irradiated at ∼80% confluency using a single 5 Gy dose administered at 2.2 Gy/min in a Mark I irradiator (J. L. Shepherd). This radiation dose was chosen based on previously established dosing ranges in studies using similar glioma cell lines [33]–[35].

### Colony formation assays

Anchorage-independent growth for the U87, U87-luc, GL261 and 0308 cell lines was assayed by the ability to form colonies in soft agar. Bottom layer agar was prepared in the appropriate medium for the cell line being assayed plus 0.6% Seakem GTG agarose (Lonza) in 6-well tissue culture plates. 3000–6000 cells (depending on cell type) were resuspended in appropriate medium containing 0.3% agar and added to each well to create the top soft agar layer. Cells were then incubated at 37°C for 3–4 weeks to allow for growth of colonies. Colonies were subsequently washed with PBS, fixed for 30 minutes with a 4% PFA solution, and visualized using an inverted light microscope. Anchorage-dependent growth of normal human astrocytes was assayed by the ability to form colonies on a tissue cultured-treated 6-well plate. 500 cells were resuspended in appropriate base medium, added to each well, and incubated at 37°C for 7 days to allow for growth of colonies. Colonies were subsequently washed with PBS and then fixed/stained with a 4% PFA/0.05% crystal violet solution prior to visualization by microscopy. All experiments were repeated using at least three independent cell samples plated in triplicate. Statistical analyses of clonogenic survival results for all cell lines and conditions were conducted on raw data values, but are displayed as a percentage of the corresponding negative control group to allow for ease of comparison among cell lines.

### Plasmid transfection

U87 cells were transiently transfected with either an empty vector (pcDNA3) or a HA-tagged HIF-1α coding construct (HA-HIF1α-pcDNA3) (Addgene, plasmid 18949) using the FuGENE6 transfection reagent (Promega) according to the manufacturer’s protocol. Transfections were timed to allow for maximal transcriptional activity during *in vitro* reoxygenation and radiation treatments.

### Western blotting

Cells were harvested at the appropriate times to match *in vitro* radiation treatment time points. Nuclear lysates were prepared in buffer containing 20 mM HEPES (pH 7.9), 25% (v/v) glycerol, 0.4 M KCl, 1.5 mM MgCl_2_, 0.2 M EDTA, supplemented with 10 mM Na_3_VO_4_, 5 mM NaFl, 5 mM DTT, 0.2 mM PMSF, and Complete Protease Inhibitor Cocktail (Santa Cruz). Nuclear protein was obtained from cells after centrifugation at 25,000×g for 30 min at 4°C. Protein concentrations were measured using Coomassie Plus Reagent (Thermo Scientific). Nuclear lysates were run on a 10% SDS-PAGE gel and electrophoretically transferred to a PVDF Immobilon membrane (Millipore). Blots were then blocked for 1 hr in blocking buffer (5% milk, 5% BSA, 2.5% normal goat serum, 0.05% Tween20, 0.02% sodium azide in TBS) and probed for nuclear proteins of interest using rabbit primary antibodies against HIF-1α (Novus Biologicals, 1∶4000) and lamin A/C (Cell Signaling Technologies, 1∶500) in 0.1x blocking buffer overnight at 4°C. After primary antibody incubation, blots were washed 4×10 min in TBS-Tween20 and incubated with a HRP-conjugated goat anti-rabbit secondary antibody (Vector Labs, 1∶25,000) in 0.1x blocking buffer at room temperature for two hours. Blots were then washed 4×10 min in TBS-Tween20 and bands were detected using SuperSignal West Dura ECL substrate (Thermo Scientific) according to the manufacturer’s protocol. Blots were imaged with a Chemidoc XRS+ Imaging System (Bio-Rad). Image analysis was carried out using Image Lab software (Bio-Rad). A representative Western blot, probed with loading control antibodies for the nuclear and cytosolic fraction-specific proteins lamin A/C (Cell Signaling Technologies, 1∶500) and β-tubulin (Santa Cruz Biotechnologies, 1∶2000), is shown in Figure S1 in order to demonstrate purity of cell fractions.

### Statistical analysis

Statistical analyses were performed using SigmaStat software. Specific statistical testing used for a given experimental design is described in the figure legends.

## Results

### Hyperoxic FiO_2_ elevates dissolved oxygen levels in brain and tumor tissue

**Figure 1:**
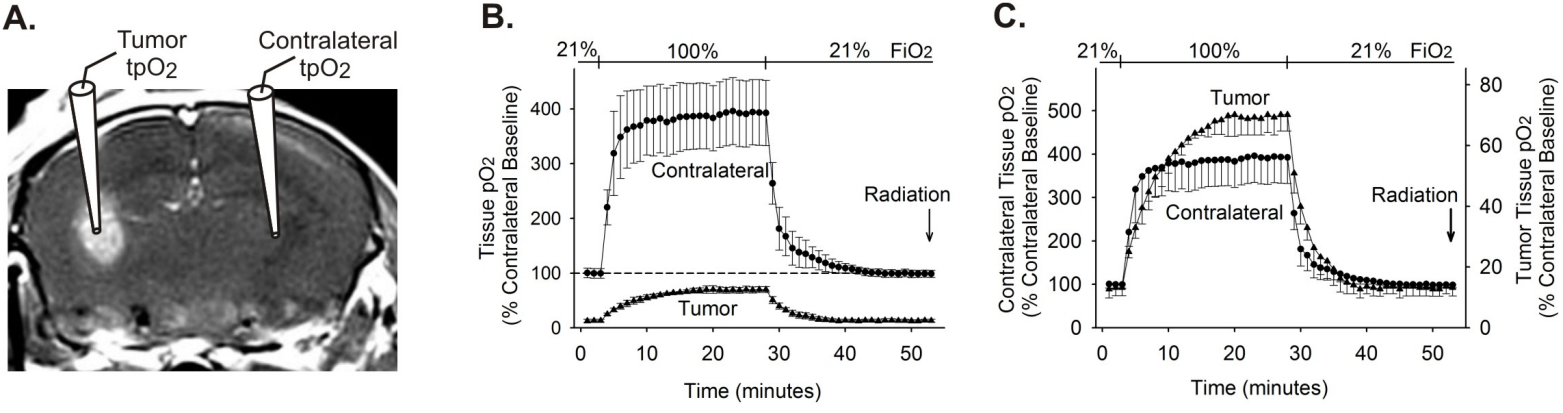
100% FiO_2_ elevates tpO_2_ in tumor and healthy brain tissue. (A) Contrast-enhanced MRI of a mouse brain that was implanted 14 days prior with U87-luc glioma cells is shown. The drawings on the MRI depict the placements of Licox probes used for measuring oxygen levels in the tumor and contralateral brain (striatum). (B and C) The time courses of partial tissue oxygen levels (tpO_2_) in response to modified FiO_2_ are shown for tumor (triangles) and contralateral (circles) tissue (n = 3). Oxygen measurements are displayed as a percentage of the baseline tpO_2_ levels recorded in the contralateral brain. In B, the time courses are plotted using a common ordinate, in order to show the relative levels and changes in tpO_2_ levels observed in tumor and contralateral brain. In C, the time courses for tumor and contralateral brain are shown on individual ordinates in order to highlight the difference in the rate of change of oxygen levels between the two tissues. Values shown are means and SEMs. Although radiation was not administered in this experiment, the arrow denoting time of radiation delivery provides a reference for subsequent experiments.

The *in vivo* oxygen pretreatment protocol consisted of a brief (25 min) exposure to 100% FiO_2_, followed by a return to ambient air (21% FiO_2_) for 25 min. In order to assess the impact of this pretreatment protocol on tissue oxygen levels, a Licox oxygen probe was used to measure levels of dissolved oxygen in both tumor, and contralateral healthy brain tissue of the same brain, in tumor-bearing mice at day 14 post-tumor implant (PTI) (Figure 1A). Baseline tpO_2_ was substantially higher in contralateral healthy striatal tissue as compared with tumor tissue, consistent with hypoxic conditions in the tumor environment (Figure 1B). Elevation of FiO_2_ from 21% to 100% effectively augmented tissue oxygenation in both healthy brain and tumor tissues. Though there was a delay to plateau in tumor tissue, both tissue types had reached asymptotic levels of oxygen enhancement ∼15 min after the onset of elevated FiO_2_ exposure (Figure 1B). The magnitude of the oxygen increase was substantially greater in the healthy brain tissue than in tumor tissue. Although the tumor tissue responded more slowly to hyperoxic FiO_2_, as compared with healthy brain, the percentage change in dissolved oxygen levels was somewhat greater in tumor tissue (Figure 1C). Upon cessation of 100% FiO_2_ treatment, both contralateral healthy brain tissue and hypoxic tumor tissue quickly returned to pretreatment oxygen levels (Figure 1B, C). These results indicate that the oxygen treatment paradigm effectively elevates dissolved oxygen levels in hypoxic tumor tissue to near-normoxic levels, and then allows this elevated oxygen to dissipate prior to radiation treatment. These findings also demonstrate that normal brain tissue reaches hyperoxic conditions during 100% FiO_2_ treatment and returns to baseline prior to radiation.

### Hyperoxic pretreatment of tumor tissue improves survival and slows intracranial tumor growth

**Figure 2:**
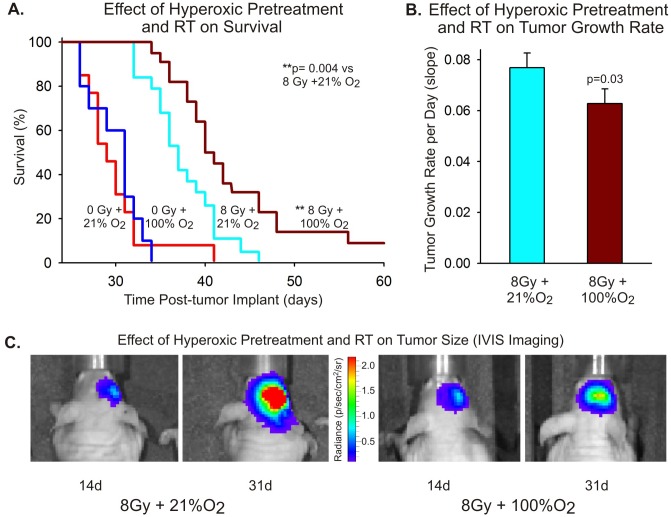
Hyperoxic pretreatment improves survival and slows tumor growth. (A) Surviving fractions of nude mice implanted with U87-luc cells are shown in a Kaplan-Meier plot for animals receiving: no treatment (0 Gy+21%O_2_, n = 13), hyperoxic pretreatment without radiation (0 Gy+100%O_2_, n = 10), radiation alone (8 Gy+21%O_2_, n = 19), and hyperoxic pretreatment with radiation (8 Gy+100%O_2_, n = 22). Statistical significance was calculated using a Log-Rank (Mantel-Cox) test. (B) A bar graph shows tumor growth rates for animals in the 8 Gy+21%O_2_ group and the 8 Gy+100%O_2_ group. Tumor size was assessed by IVIS imaging *in vivo* performed every 4–5 days. The log of the radiance from up to eight IVIS time points (day 14 to day 46 PTI, depending on individual survival) for each animal was used to perform linear regression analyses of tumor growth. Calculated slopes from these linear regressions were then used to determine average tumor growth rate per day for each animal from each treatment group. Statistical significance was calculated using t-test with Welch’s correction for unequal variance. Error bars are SEMs. (C) IVIS images taken at day 14 and day 31 PTI for two animals, one from the 8 Gy+21%O_2_ (left) and one from the 8 Gy+100%O_2_ (right) groups. The scale bar for IVIS radiance is shown between the images for the two animals. Note that the IVIS signal in these images does not directly correspond to tumor size. A low luminescence signal threshold was used to permit comparison between time points.

In order to evaluate the efficacy of hyperoxic pretreatment as an adjuvant to radiation therapy for GBM, nude mice with U87-luc tumors were assigned to one of four experimental groups after preliminary tumor imaging on day 14 PTI: untreated (0 Gy+21% O_2_, n = 13), hyperoxic pretreatment without radiation (0 Gy+100% O_2_, n = 10), radiation alone (8 Gy+21% O_2_, n = 19), and hyperoxic pretreatment with radiation (8 Gy+100% O_2_, n = 22). Tumor growth was monitored using IVIS imaging every four to five days during the survival period of each animal. Animals that received transient hyperoxic pretreatment prior to radiotherapy showed a significant improvement in survival as compared with animals that received radiation alone (Figure 2A; Table S1). As measured by IVIS, tumor growth was slowed significantly in animals receiving hyperoxic pretreatment prior to radiation (Figure 2B; Table S2; Figure S2). Representative IVIS images from animals in the 8 Gy+21% O_2_ group and 8 Gy+100% O_2_ group are shown in Figure 2C. The animal that received hyperoxic pretreatment exhibited less luciferase activity at day 31 PTI compared with the animal that did not receive pretreatment. It should be noted that while IVIS measurements provide an index of tumor size that is useful for monitoring tumor growth, IVIS luminescence does not reflect precisely the anatomical volume of the tumor. Luminescence signal appears widespread in the Day 31 PTI images because of the low signal threshold utilized for comparison among early and late time points.

### Transient reoxygenation sensitizes glioma cells to radiation and temporarily reduces nuclear HIF-1α accumulation in *in vitro* models of tumor hypoxia

**Figure 3:**
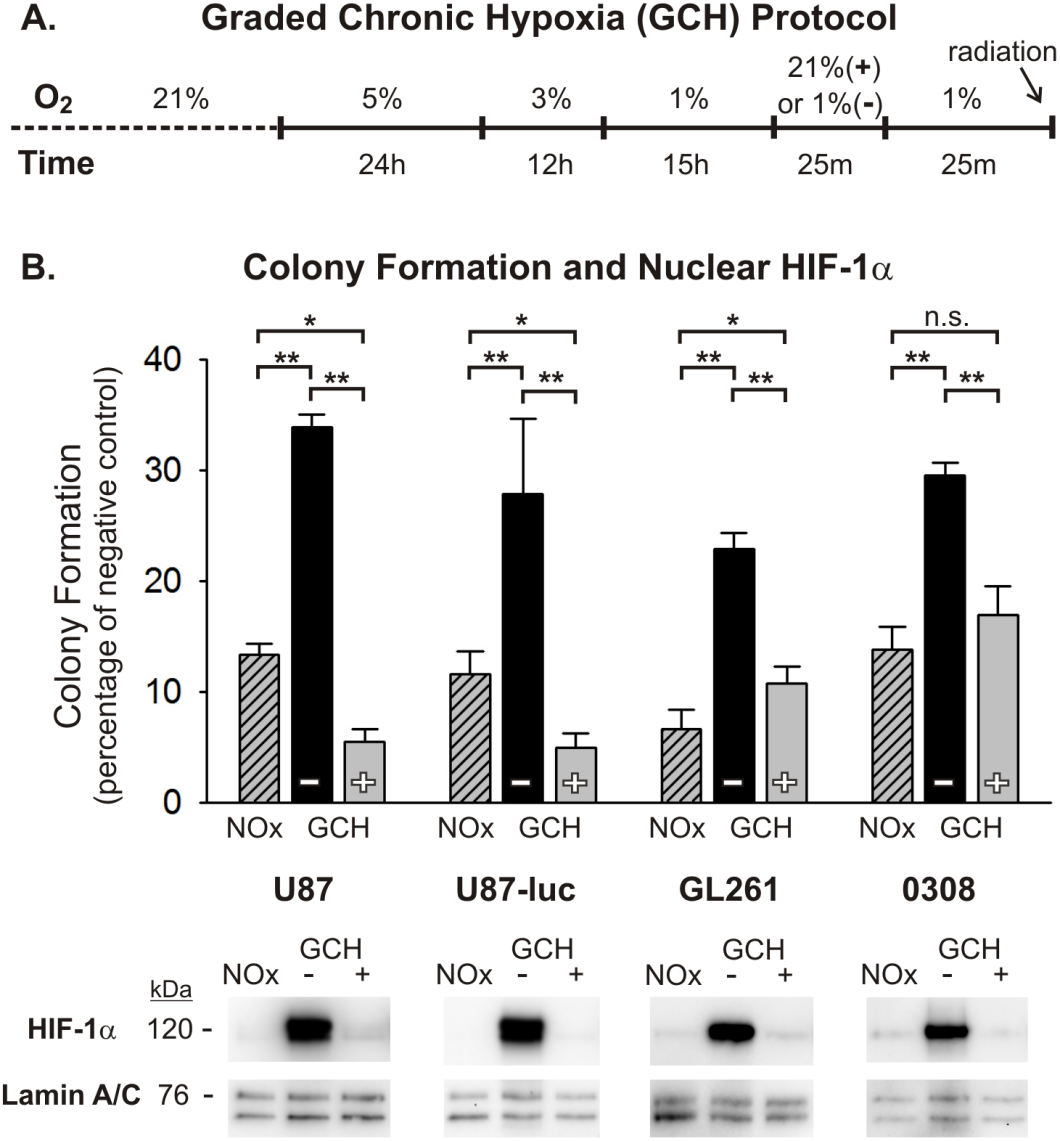
Normoxic pre-treatment sensitizes glioma cells to radiation after graded chronic hypoxic (GCH) exposure. (A) The graded chronic hypoxia (GCH) protocol is shown, depicting the timing and severity of hypoxic exposure to four cell lines. Cells either remain in a continuous hypoxic environment (–) or are transiently (25 min) exposed to normoxia 25 min prior to radiation (+). Continuously normoxic cells (NOx) were irradiated as a positive control. (B) The results of anchorage-independent colony forming assays are shown for U87, U87-luc, GL261 glioma cells and 0308 GSCs after 5 Gy radiation exposure under varying oxygen conditions. To allow for ease of comparisons among cell types, raw values are expressed as a percentage of the corresponding cell type’s negative (non-irradiated) control and the means and SEMs are plotted. Each result represents at least three independent samples, plated in triplicate. Holm-Sidak comparisons for multiple groups were used for statistical comparisons of raw values (*p<0.05, **p<0.01). Also shown are Western blots of nuclear HIF-1α at the time of irradiation for each cell type. Corresponding Western blots of lamin A/C are shown as a loading control.

**Figure 4:**
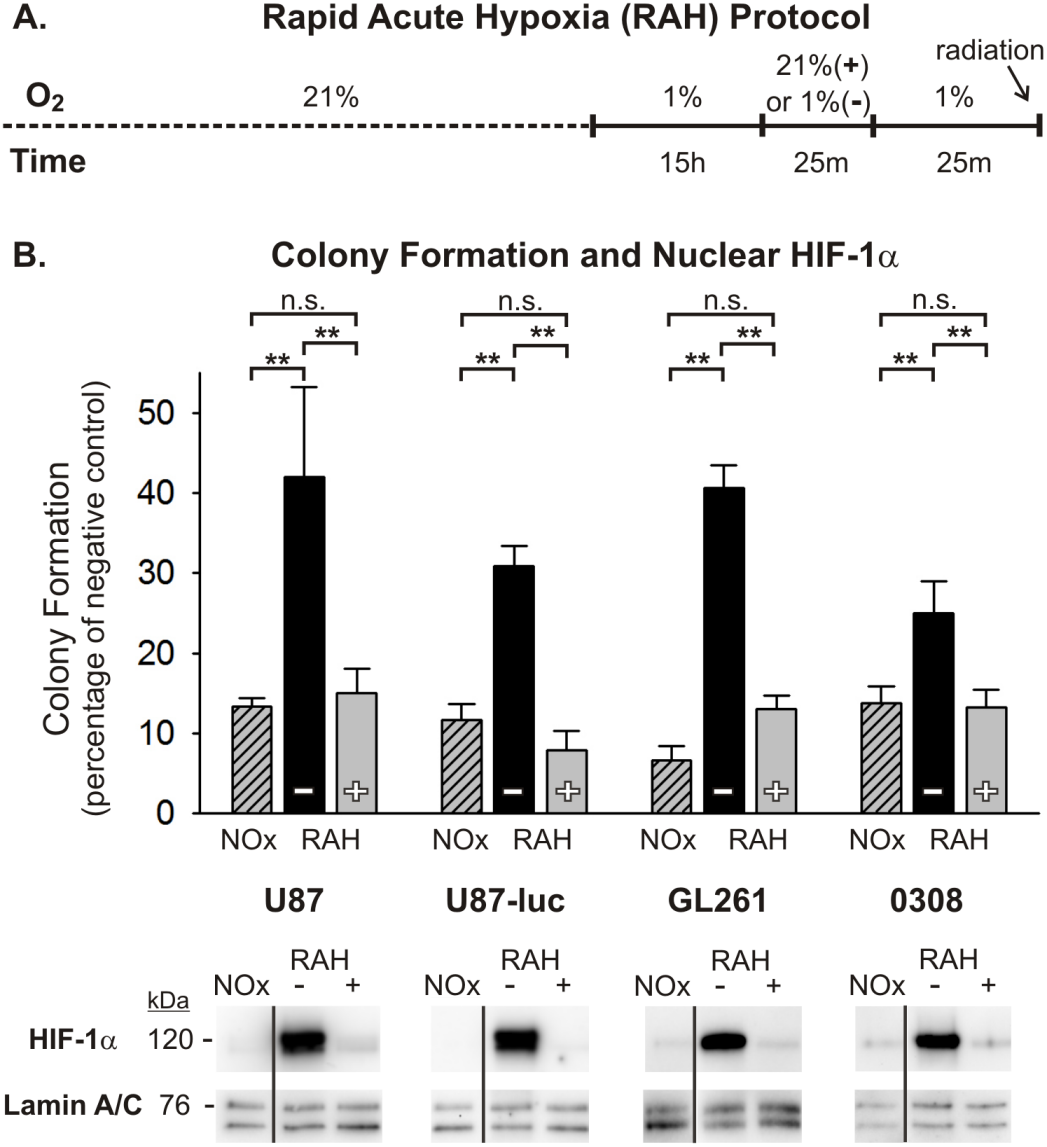
Normoxic pretreatment sensitizes glioma cells to radiation after rapid acute hypoxic (RAH) exposure. (A) The rapid acute hypoxia (RAH) protocol is shown depicting the timing and severity of hypoxic exposure. Cells either remain in a continuous hypoxic environment (–) or are transiently (25 min) exposed to normoxia 25 min prior to radiation (+). Continuously normoxic cells (NOx) were irradiated as a positive control. (B) The results of anchorage-independent colony forming assays are shown for U87, U87-luc, GL261 glioma cells and 0308 GSCs after 5 Gy radiation exposure under varying oxygen conditions. To allow for ease of comparisons among cell types, raw values are expressed as a percentage of the corresponding cell type’s negative (non-irradiated) control and the means and SEMs are plotted. Each result represents at least three independent samples, plated in triplicate. Holm-Sidak comparisons for multiple groups were used for statistical comparisons of raw values (**p<0.01). Also shown are Western blots of nuclear HIF-1α at the time of irradiation for each cell type. Corresponding Western blots of lamin A/C are shown as a loading control. All lanes shown that are non-adjacent to the negative control (NOx) are denoted with a separating black line.

In order to further assess the role of transient oxygen elevation in the enhanced radiotherapeutic vulnerability seen in tumors *in vivo*, we utilized two simplified *in vitro* models of tumor hypoxia. The glioma microenvironment represents a heterogeneous mixture of oxygen conditions for tumor cells that arise from several intra-tumor vascular factors, many of which stem from immature and incompetent blood vessels. Consequently, glioma cells can be exposed to highly variable durations and severities of hypoxic stress, which has important implications regarding how these cells respond to various anti-tumor therapies, including radiation. In an effort to mimic two different hypoxic conditions in a controlled manner, a graded chronic hypoxia (GCH) protocol and a rapid acute hypoxia (RAH) protocol were used (see Figures 3A and 4A). U87, U87-luc, and GL261 glioma cells, and 0308 GSCs exposed to the GCH protocol (Figure 3A) showed a significant resistance to a 5 Gy dose of radiation under continuous hypoxic conditions compared with normoxic controls, as demonstrated by anchorage-independent colony forming assays (Figure 3B). When these cells were transiently exposed to normoxic levels of oxygen for 25 minutes and then returned to severe hypoxia (1% O_2_) for 25 minutes prior to radiation exposure, clonogenicity was significantly impaired in all assayed cell lines and was below the clonogenic ability of cells irradiated under normoxic conditions in the U87 and U87-luc cell lines (Figure 3B; Figure S3). Nuclear fractions from all cell lines and conditions at the time of radiation exposure were assessed using Western blot analysis with an anti-HIF-1α antibody. Results from these experiments demonstrated dramatically elevated accumulation of HIF-1α protein in the nuclei of these cells under the GCH protocol. Moreover, following brief reoxygenation and return to hypoxia for 25 minutes, this nuclear accumulation of HIF-1α protein was reduced substantially (Figure 3B). Similar clonogenic results were seen in cells exposed to the RAH protocol (Figure 4A) and treated with a 5 Gy dose of radiation. Transient reoxygenation prior to radiation produced a significant radiosensitization of each cell line, although the clonogenic abilities of the U87 and U87-luc cells were not impaired to the extent that they were following the GCH protocol (Figure 4B; Figure S4). As seen in cells exposed to GCH, there was significant accumulation of HIF-1α protein in the nuclei of cells under the RAH protocol, and this nuclear accumulation was reduced substantially following reoxygenation and return to hypoxia.

### Transient reoxygenation-induced glioma cell sensitization persists for up to 3 hours after return to hypoxia

**Figure 5:**
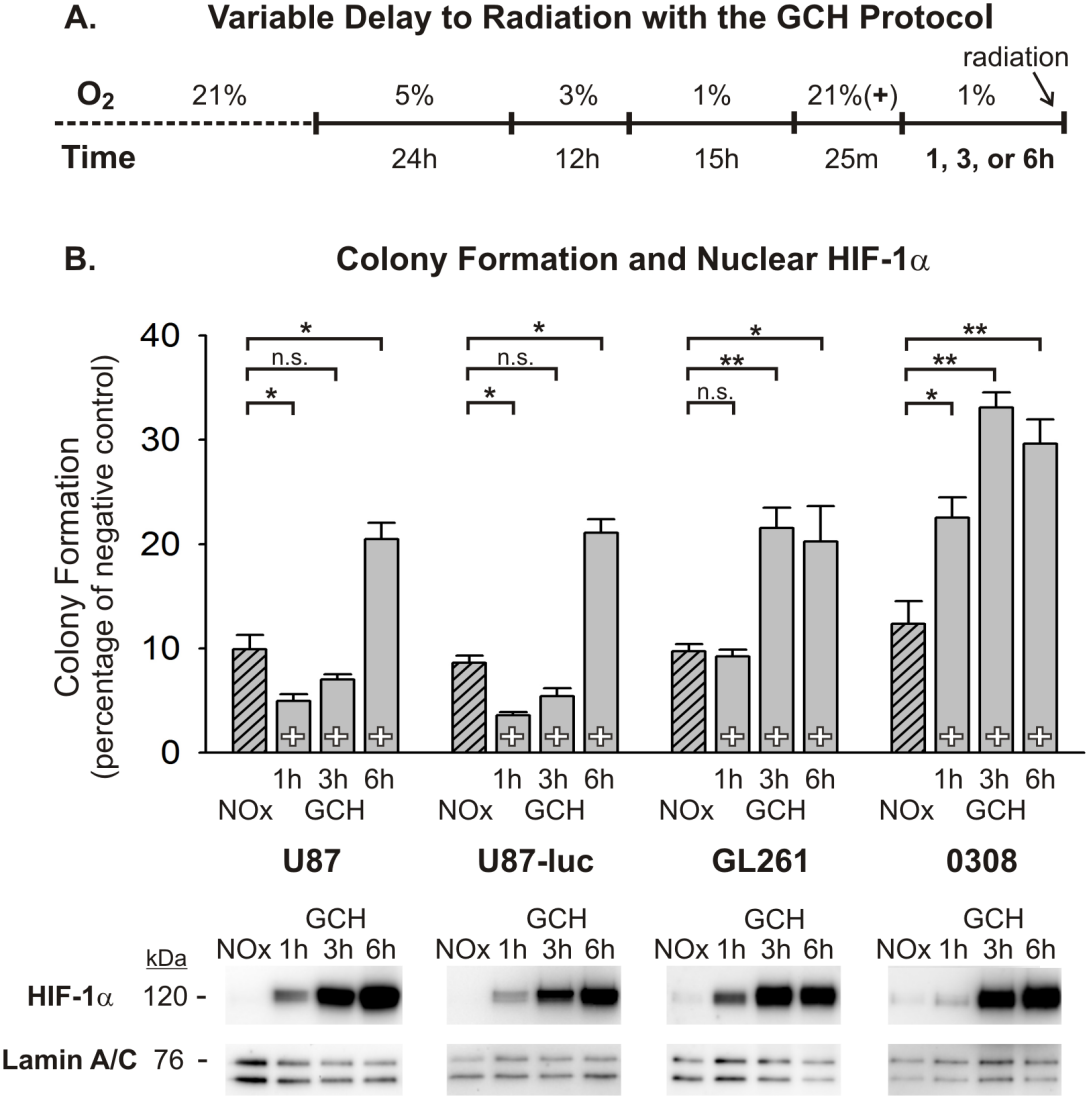
The duration of enhanced radiosensitivity after normoxic pretreatment differs among cell lines undergoing Graded Chronic Hypoxia. (A) The GCH protocol is shown with variable delays to radiation after normoxic pretreatment. After GCH, all cells are transiently (25 min) exposed to normoxia for 25 min (+) and are then returned to severe hypoxia (1% O_2_) for 1, 3, or 6 hours prior to radiation. Continuously normoxic cells (NOx) were irradiated as a positive control. (B) Results from anchorage-independent colony forming assays indicate that the decay of enhanced radiosensitivity differs among cell lines. To allow for ease of comparisons among cell types, raw values are expressed as a percentage of the corresponding cell type’s negative (non-irradiated) control and the means and SEMs are plotted. Each result represents at least three independent samples, plated in triplicate. Holm-Sidak comparisons for multiple groups were used for statistical comparisons of raw values (*p<0.05, **p<0.01). Also shown are Western blots of nuclear HIF-1α at the time of irradiation for each cell line. Corresponding Western blots of lamin A/C are shown as a loading control.

**Figure 6:**
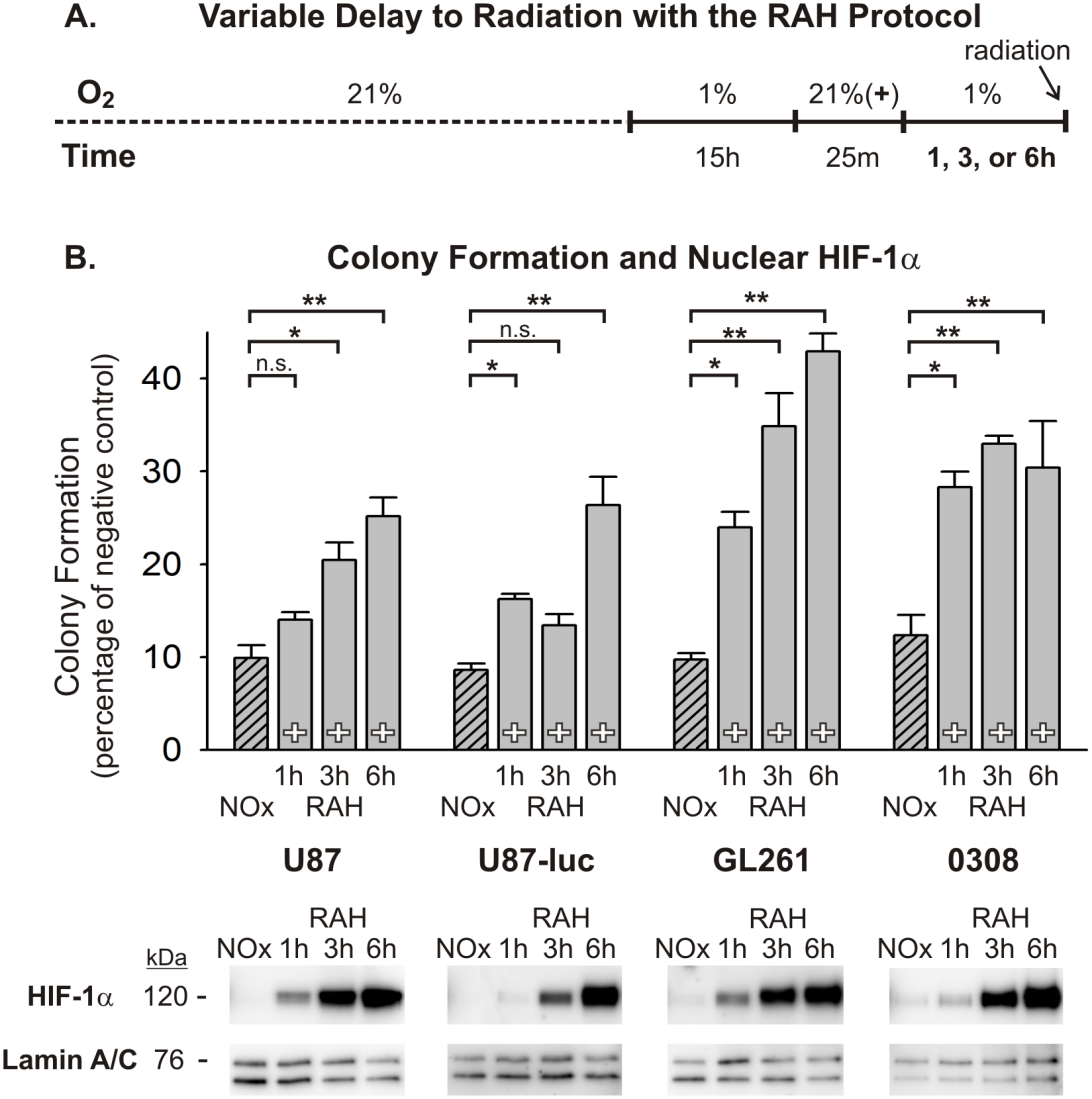
The duration of enhanced radiosensitivity after normoxic pretreatment of cells undergoing Rapid Acute Hypoxia. (A) The RAH protocol is shown with variable delays to radiation after normoxic pretreatment. In this protocol all cells are transiently (25 min) exposed to normoxia for 25 min (+) and then returned to severe hypoxia (1% O_2_) for 1, 3, or 6 hours prior to radiation. Continuously normoxic cells (NOx) were irradiated as a positive control. (B) Results from anchorage-independent colony forming assays indicate that the decay of enhanced radiosensitivity for cells in the RAH protocol is generally more rapid than that observed for cells in the GCH protocol. To allow for ease of comparisons among cell types, raw values are expressed as a percentage of the corresponding cell type’s negative (non-irradiated) control and the means and SEMs are plotted. Each result represents at least three independent samples, plated in triplicate. Holm-Sidak comparisons for multiple groups were used for statistical comparisons of raw values (*p<0.05, **p<0.01). Also shown are Western blots of nuclear HIF-1α at the time of irradiation for each cell line. Corresponding Western blots of lamin A/C are shown as a loading control.

The time frame over which glioma cells sustain this increased radiosensitivity after transient oxygenation was investigated by irradiating cells at one of three post-oxygenation time points. Glioma cells subjected to the GCH protocol and then returned to 1% O_2_ for 1, 3, or 6 hours prior to 5 Gy radiation treatment (see Figure 5A) demonstrated oxygen-induced radiosensitization persisting for at least one hour after returning to hypoxic conditions. U87 and U87-luc cell clonogenic capacities remained impaired at 3 hours post-reoxygenation before these cells began to recover resistance at 6 hours post-reoxygenation. GL261 clonogenicity recovered more quickly, remaining impaired at 1 hour, but recovering by 3 hours post-reoxygenation. 0308 GSCs proved more refractory to the effects of transient reoxygenation, reestablishing significant radiation resistance at 1 hour post-reoxygenation (Figure 5B; Figure S5). Western analysis of nuclear lysates revealed a recovery of HIF-1α accumulation that begins prior to the recovery of cellular resistance to radiation in U87, U87-luc, and GL261 cells, but corresponds temporally with recovery of radiation resistance in 0308 GSCs (Figure 5B). The same post-reoxygenation time points for radiation (1, 3, 6 hours) were also tested using the RAH protocol (see Figure 6A). In general, each of the cell lines appears to recover resistance to radiation exposure more quickly as compared with cells that were subjected to the GCH protocol. U87, U87-luc, and GL261 clonogenic capacity improved progressively from 1 hour to 6 hours post-reoxygenation; 0308 GSCs almost fully recover radiation resistance by 1 hour post-reoxygenation (Figure 6B; Figure S6). The timing of recovery of nuclear HIF-1α accumulation following the RAH protocol, as shown by Western blot, closely corresponds with recovery of cellular resistance to radiation in all cell lines (Figure 6B).

### Overexpression of HIF-1α rescues radiation resistance in cells exposed to RAH, but not GCH

**Figure 7:**
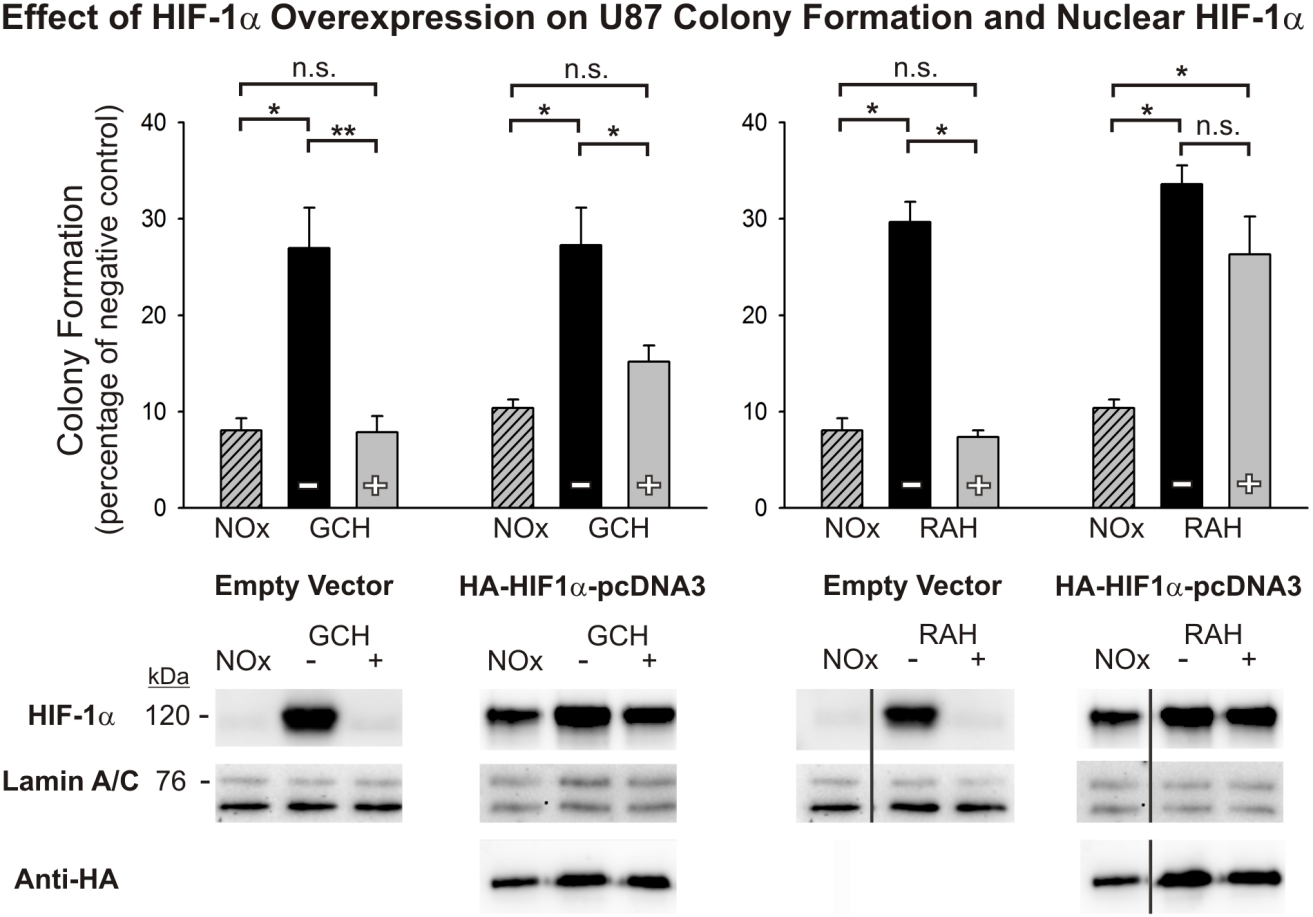
HIF-1α overexpression rescues oxygen-induced radioresistance in RAH-treated cells, but not GCH-treated cells. Results are shown for the anchorage-independent colony forming assays for U87 cells transfected with either an empty vector or HIF-1α expression vector and then exposed to GCH or RAH protocols without (–) or with (+) reoxygenation. Continuously normoxic cells (NOx) were irradiated as a positive control. To allow for ease of comparisons among conditions, raw values are presented as a percentage of that cell type’s negative (non-irradiated) control and the means and SEMs are plotted. Each result represents at least three independent samples, plated in triplicate. Holm-Sidak comparisons for multiple groups were used for statistical comparisons of raw values (*p<0.05, **p<0.01). Western blotting analysis of nuclear HIF-1α at the time of irradiation is shown for each cell type below clonogenic results. Corresponding Western blots of lamin A/C are shown as a loading control and blots for hemagglutinin (HA) are shown below HIF-1α overexpression vector results to demonstrate transfection efficacy. All lanes shown that are non-adjacent to the negative control (NOx) are denoted with a separating black line.

In order to examine the effect of nuclear accumulation of HIF-1α in the radiation resistance of glioma cells following transient reoxygenation *in vitro*, U87 cells were transfected with either a HIF-1α-expressing construct (HA-HIF1α-pcDNA3) or an empty vector (pcDNA3). Cells were then exposed to the GCH or RAH protocol (see Figures 3 and 4), without or with reoxygenation, and assayed for clonogenicity following 5 Gy radiation exposure. Cells transfected with an empty vector and exposed to GCH(+) prior to radiation demonstrate enhanced radiation vulnerability comparable to that of non-transfected cells, when assessed with the colony forming assay. While HIF-1α overexpression in GCH(+) treated cells provided for a trend towards increased clonogenicity compared with over-expressing normoxic controls, this change did not achieve statistical significance. Similarly, RAH(+) cells transfected with an empty vector mirrored the enhanced vulnerability of non-transfected cells. However, overexpression of HIF-1α in RAH(+) treated cells rescued radiation resistance and abolished the effects of transient reoxygenation (Figure 7; Figure S7). Western blot analyses showed substantial nuclear accumulation of HIF-1α in cells transfected with the HIF-1α over-expression construct under all experimental conditions. Most notably, high levels of nuclear HIF-1α protein were maintained in U87 cells following transient reoxygenation in both the GCH and RAH protocols, and were present at the time of radiation exposure (Figure 7). These results suggest that HIF-1α may play a more critical role in the resistance to hyperoxic pretreatment of glioma cells exposed to acute levels of hypoxia than it does in cells maintained under more chronic hypoxic conditions.

### Transient hyperoxia does not sensitize normal human astrocytes to radiation

**Figure 8:**
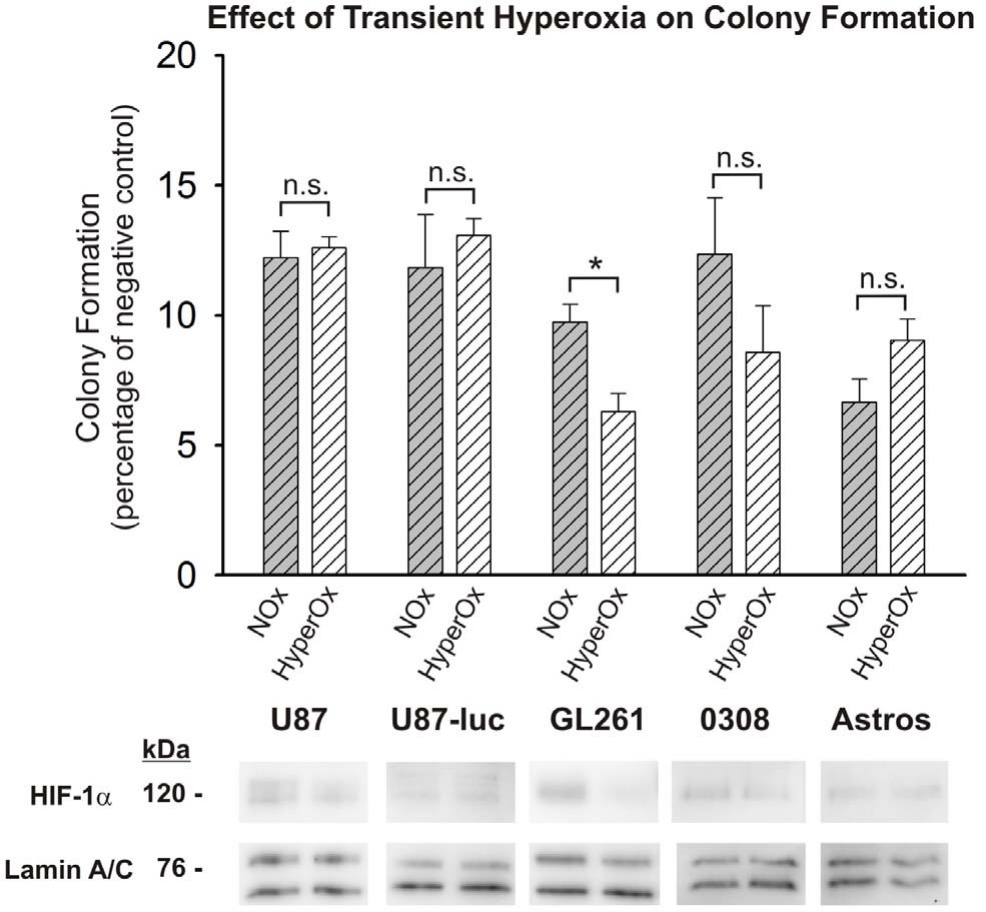
Transient hyperoxia does not sensitize normal human astrocytes to radiation. Results are shown for the colony forming assays for all previously assayed cell lines and a normal human astrocyte cell line (Astro). Cells were continuously maintained under normoxic conditions (NOx) or exposed to 25 min of hyperoxia (50% O_2_) and then returned to normoxic conditions for 25 min (HyperOx) before being treated with a 5 Gy dose of radiation. To allow for ease of comparisons among cell types, raw values are expressed as a percentage of the corresponding cells type’s negative (non-irradiated) control and the means and SEMs are plotted. Each result represents three independent samples, plated in triplicate (*p<0.05, Student’s t-test). Also shown are Western blots of nuclear HIF-1α at the time of irradiation for each cell line. Corresponding Western blots of lamin A/C are shown as a loading control.

As demonstrated by the tissue oxygen probe results presented in Figure 1, the administration of 100% FiO_2_ dramatically elevates oxygen levels in healthy brain tissue. In order to investigate the possibility that this transient hyperoxic treatment could sensitize previously normoxic brain tissue, we exposed normal human astrocytes to 25 minutes of hyperoxia (50% O_2_) before returning them to normoxic (21% O_2_) conditions for 25 minutes and treating them with a 5 Gy dose of radiation. Results from clonogenic assays demonstrate that hyperoxic pretreatment does not sensitize normal human astrocytes and, in fact, trended towards protecting these cells from the effects of radiation, although this effect did not achieve statistical significance (Figure 8; Figure S8). Identical hyperoxic pretreatment of the glioma cell lines and GSCs generally showed no effect on clonogenicity compared with normoxic controls in U87, U87-luc and 0308 cells (Figure 8; Figure S8). GL261 cells, however, exhibited enhanced radiosensitivity following transient hyperoxic exposure that was statistically significant, indicating a potential divergence in vulnerabilities of normal astrocytes and glioma cells following pretreatment with hyperoxia, even when baseline conditions are normoxic. Nuclear fractions from all cell lines at the time of radiation following both normoxic and hyperoxic exposure were assessed using Western blot analysis with an anti-HIF-1α antibody. No discernable differences in nuclear HIF-1α accumulation were observed in any of the cell lines following hyperoxic exposure, when compared with cells maintained under normoxia.

## Discussion

Recent advances in our understanding of the cellular and molecular characteristics of gliomas have provided hope for developing promising alternatives to the traditional mainstays for treatment of this lethal disease [36]–[38]. Nonetheless, the vast majority of patients still receive surgical resection, radiotherapy, and chemotherapy as a standard of care. Even with this aggressive approach to treatment, tumor recurrence is universal, after which continued radiation and chemotherapy are the primary treatment modalities [39], [40]. The presence of hypoxic niches within the solid tumor microenvironment represents a serious challenge to the success of standard therapies. It is therefore of critical importance to identify new and effective ways to optimize the targeting of hypoxic glioma cells. The present study reveals a novel means by which a well-studied non-invasive, non-pharmocological adjuvant sensitizes glioma cells to radiotherapy. Transient elevation of tissue oxygen via normobaric hyperoxia provides a window of enhanced glioma cell vulnerability to ionizing radiation that persists well after tumor tissue has returned to hypoxic conditions.

The ability of ionizing radiation to damage cells is related to the concentration of molecular oxygen present in the tissue at the time of radiation [41]. The low level of molecular oxygen in hypoxic tumor environments renders radiotherapy less effective than would be achieved under normoxic conditions. Consequently, considerable effort has been directed toward increasing the levels of molecular oxygen in the tumor region during radiotherapy [20], [21], [23]. However, elevating tissue oxygen introduces the risk of increasing the vulnerability of radiation injury to healthy tissue. As shown here and elsewhere [42]–[44], systemic oxygen treatment creates hyperoxic conditions in normal brain tissue (Figure 1), which can place non-target cells at a higher risk for injury when radiation is administered while oxygen is elevated. In fact, we show that the magnitude of oxygen elevation in healthy tissue in response to 100% FiO_2_ is considerably greater than that observed in tumor tissue, and the rate of this increase in normal tissue is more rapid. This underscores the potential risk to healthy tissue that is present when radiation is delivered during hyperoxia. Nonetheless, the goal of elevating tumor tissue oxygenation is also achieved by increasing FiO_2_. This elevation is considerable, nearly reaching normal physiological levels within 15 minutes of the onset of hyperoxia. There has been a general consensus that the presence of elevated oxygen in tissue is necessary at the time of radiation for oxygen to sensitize radioresistant cells. However, our findings indicate that oxygen-induced radiosensitization is not completely dependent on the presence of elevated oxygen during radiotherapy. We show that there is in fact an extended window of cellular vulnerability that persists well beyond the point at which these cells return to their baseline hypoxic conditions. It is therefore reasonable to assume that other cellular or molecular modifications underlie this shift in tolerance, and that these mechanisms operate in addition to the direct radiosensitizing effects of molecular oxygen.

A key assumption regarding this shift in vulnerability is that oxygen induces persistent radiosensitivity via mechanisms that are intrinsic to the glioma cells. We tested this concept using simplified *in vitro* models of tumor hypoxia, representing two different durations and severities of hypoxic conditions. We were able to confirm, in multiple glioma cells lines, the radiosensitizing effects of transiently increasing oxygen levels. The findings show that radiosensitization is produced, at least in part, by a direct effect on glioma cells, indicating that a change in intrinsic glioma cell resistance mechanisms contributes to oxygen-induced enhanced tumor vulnerability.

Based on the critical role that the transcription factor HIF-1 is known to play in GBM progression and treatment resistance, we also assessed HIF-1α expression levels in the glioma cell lines at multiple time points following hypoxic conditions and reoxygenation exposures. HIF-1 regulates numerous genes responsible for tumor cell survival, growth, and invasiveness at the transcriptional level [17]. The HIF-1α subunit is over-expressed in many human cancers, due in large part to extensive areas of tumor hypoxia and loss-of-function and gain-of-function mutations in genes regulating HIF-1α [45]. This contributes to the aggressiveness and treatment-resistance of tumors through the promotion of anti-apoptotic genes (e.g. BCL-xL), and inhibition of pro-apoptotic genes (e.g. IAP-2) [46], [47]. The availability of HIF-1α to act on target responsive elements when heterodimerized with HIF-1β is dependent upon its nuclear translocation. Western blot analyses of nuclear fractions from each of the cell lines used in this study demonstrated elevated nuclear HIF-1α accumulation under hypoxic conditions. Notably, transient reoxygenation of these cells following both the GCH and the RAH protocols reduces nuclear HIF-1α protein expression to near pre-hypoxic levels. And, these decreased levels of HIF-1α persist for at least one hour after the cells have returned to severe hypoxic conditions, a phenomenon that could potentially be explained by the persistence of elevated HIF-a-prolyl-hydroxylase levels in cells exposed to prolonged hypoxia. Marxsen et al [48] demonstrate that accumulation of HIF-1α in cells during prolonged hypoxia leads to a concomitant accumulation of the HIF-a-prolyl-hydroxylases, PHD2 and PHD3. Upon reoxygenation, elevated levels of PHD2 and PHD3 facilitate the rapid degradation of HIF-1α, and while PHD protein levels begin to decline soon after reoxygenation, there persists a relative elevation in PHDs for up to 24 hours post-reoxygenation. Based on this evidence, it is reasonable to suggest that the refractory accumulation of HIF-1α in the nucleus of glioma cells following reoxygenation and subsequent return to hypoxia in our models is at least partly due to an upregulation of PHD2 and PHD3 activity.

The recovery of high HIF-1α nuclear expression after oxygen treatment corresponds with the recovery of radiation resistance in cells subjected to RAH, but to a lesser extent in cells subjected to GCH. Interestingly, when we overexpressed HIF-1α in U87 cells exposed to GCH or RAH, the reduction of nuclear HIF-1α resulting from transient reoxygenation was abated. Artificially maintaining HIF-1α at high levels during and immediately following reoxygenation rescued radioresistance in RAH-treated, but not GCH-treated, cells. This suggests that the specific hypoxic history of each cell within a given tumor dictates the characteristics of the cellular response to transient reoxygenation and the subsequent period of radiosensitization. This concept speaks to the heterogeneity of the tumor microenvironment and the complexity of developing therapeutics aimed at targeting these treatment-resistant tumor cells.

From these results, it is reasonable to speculate that a temporary reduction in nuclear HIF-1 activity contributes to a subsequent repression of the pro-survival mechanisms normally being maintained in certain hypoxic tumor cells. This could help explain the increased vulnerability of transiently reoxygenated cells to ionizing radiation under hypoxic conditions compared with cells that have not been exposed to reoxygenation. The reported roles played by the HIF-1α subunit and HIF-1 transcription factor within the context of the hypoxic response in both healthy cells and cancer cells are very complex [12], [49]. The hypoxia models used in our studies demonstrate a difference in the influence of HIF-1α on cell vulnerability following reoxygenation and radiation after chronic versus acute hypoxic exposure. Further studies are needed in order to elucidate how such a temporary shut down of HIF-1 activity might impact the expression of its myriad downstream targets, and how alterations in the availability of these gene products might contribute to the extended vulnerability observed in oxygen pretreated glioma cells. Based on previous investigations into the effects of silencing HIF-1α on radiation survival outcomes in U87 and U251 glioma cells [50], [51], we know that enhanced radiotherapeutic efficacy following downregulation of HIF-1 can result from delayed cell cycle progression and increased incidence of mitotic catastrophe and apoptosis. It will be important to investigate whether the temporary absence of nuclear HIF-1α and attendant increase in hypoxic cell vulnerability to radiation after transient oxygen exposure in our models results in a similar range of cellular and molecular changes that lead to multiple cell fates.

The potential clinical relevance of oxygen-induced tumor cell vulnerability is that it holds the possibility to be effective by selectively increasing the sensitivity of treatment-resistant tumor cells to radiation, while not sensitizing non-target, healthy brain tissue. Many preclinical and clinical investigations have attempted to use elevated tumor oxygenation during radiotherapy in efforts to sensitize radio-resistant glioma tissue [20], [21], [23], [52]. A major drawback to these approaches, however, is that while increasing oxygen levels in the blood can elevate oxygen levels in hypoxic tumor tissue, it also produces hyperoxic levels of oxygen in the surrounding healthy tissue. The work conducted by Kohshi et al [52] on hyperbaric oxygen (HBO) therapy as a radiotherapeutic adjunct clearly demonstrates this point. Even with the use of more focused radiation techniques such as stereotactic radiotherapy for the treatment of brain tumors, it remains difficult to fully compensate for the irregular tumor margins present in high-grade gliomas, making collateral brain tissue damage an unfortunate reality. In the Kohshi et al study, the delay between HBO therapy and radiation was intentionally kept very short (7 minutes) in order to administer the radiotherapy during the period of elevated tissue oxygenation [53]. The resulting symptomatic radionecrosis in a significant portion of enrolled patients (28%) emphasizes the need for alternative approaches when combining these two treatment modalities.

In contrast to this previous work, the approaches presented herein transiently elevate tissue oxygenation, but allow for tissue oxygen to return to baseline levels in both healthy and tumor tissue prior to treatment. Thus, the risk posed to eloquent tissue by elevated molecular oxygen at the time of radiation exposure is minimized. In fact, it is conceivable that hyperoxic preconditioning could actually reduce the vulnerability of healthy normoxic tissue [54]. In order to investigate this possibility, we exposed normal human astrocytes maintained under normoxic conditions to transient hyperoxia and then returned them to normoxia before exposing them to radiation. Importantly, transient hyperoxia did not sensitize normal astrocytes to radiation. Rather, there was a trend toward desensitizing the astrocytes, as suggested by a slight increase in the clonogenic capacity of these cells. In contrast, GL261 cells were sensitized to radiation following transient hyperoxia, while the radiosensitivity of the other glioma cell lines was not affected by hyperoxic exposure. These results suggest that transient oxygen pretreatment prior to radiation therapy may allow for a divergence in the vulnerabilities of hypoxic tumor vs. healthy brain tissue that optimizes the injury produced in the target tumor tissue while limiting injury to normal tissue. The possibility of divergent tissue vulnerabilities underscores the potential benefit of using this type of adjuvant oxygen therapy during the course of radiation treatment in patients with GBM.

It is worth noting that the radiation treatment protocol used in our study employed a moderate, single dose of whole-head radiation. It will be valuable for future studies to evaluate the impact of oxygen pretreatment in conjunction with multiple radiation treatments using a fractionated dose protocol. In addition, other variants of hypoxic stress, such as cycling and intermittent hypoxia, are known to occur in solid tumors [33], [55]–[57], and certain courses of hypoxia and reoxygenation have been shown to aid in tumor treatment resistance and progression via ROS signaling and regulation of stress granules [58]. It will therefore be important to assess the cellular and molecular impacts of transient oxygenation treatment for glioma cells under various forms of hypoxic challenge, as well as to evaluate the response of other important cell populations, such as endothelial cells, both in the tumor microenvironment and in the healthy brain. Additional investigations of this type will aid in our understanding of the dynamics of oxygen-induced glioma cell radiosensitization, which may lead to the implementation of a novel, safe, and non-invasive approach for selective tumor cell treatment.

## Supporting Information

Figure S1 **Confirmation of purity of nuclear and cytosolic fractions.** In order to demonstrate purity of nuclear and cytosolic fractions generated from whole cell lysates, representative Western blot images are shown. Nuclear (Nuc) and cytosolic (Cyto) fractions from the U87 NOx sample were subjected to SDS-PAGE and Western analysis. Blots were probed using appropriate loading control antibodies for nucleus-specific (lamin A/C) and cytosol-specific (β-tubulin) proteins. Lamin A/C was highly enriched in the nuclear fraction, while β-tubulin was not detected. In contrast, β-tubulin was enriched in the cytosolic fraction, while lamin A/C was not detected.
(JPG)

Figure S2 **Tumor growth plots: linear regressions for individual animals.** Linear regression plots of tumor growth over time are shown for animals in the 8 Gy+21% O_2_ and 8 Gy+100% O_2_ treatment groups. Individual data points represent the log of tumor radiance, as assessed by IVIS at each tumor measurement time point. Slope values for tumor growth slope were generated from these linear regression plots and are shown in Table S2.
(JPG)

Figure S3 **Effect of graded chronic hypoxia (GCH) on clonogenic survival.** Raw clonogenic data, expressed as the number of surviving colonies, are shown for cells exposed to radiation under continuous normoxia (NOx), graded chronic hypoxia without reoxygenation (GCH−), or graded chronic hypoxia with reoxygenation (GCH+). Each data point (solid circle) represents the average of three replicates within a given experiment. Three independent experiments were run for each condition and the average value for the three experiments is shown as a red triangle. Statistical assessments for group differences used the Holm-Sidak test for multiple comparisons. The statistical comparisons performed on the raw data are presented in Figure 3B. Note that the data presentation for average group values in Figure 3B is normalized as a percentage of the average clonogenic survival of the negative control for a given cell type. Normalization of the data in this manner allows for presentation on a common y-axis and facilitates group comparisons. The average clonogenic survival of the negative group for each cell type was: U87 = 109.92; U87-luc = 91.67; GL261 = 83.22; 0308 = 561.42.
(JPG)

Figure S4 **Effect of rapid acute hypoxia (RAH) on clonogenic survival.** Raw clonogenic data, expressed as the number of surviving colonies, are shown for cells exposed to radiation under continuous normoxia (NOx), rapid acute hypoxia without reoxygenation (RAH−), or rapid acute hypoxia with reoxygenation (RAH+). Each data point (solid circle) represents the average of three replicates within a given experiment. Three independent experiments were run for each condition and the average value for the three experiments is shown as a red triangle. Statistical assessments for group differences used the Holm-Sidak test for multiple comparisons. The statistical comparisons performed on the raw data are presented in Figure 4B. Note that the data presentation for average group values in Figure 4B is normalized as a percentage of the average clonogenic survival of the negative control for a given cell type. Normalization of the data in this manner allows for presentation on a common y-axis and facilitates group comparisons. The average clonogenic survival of the negative group for each cell type was: U87 = 109.92; U87-luc = 91.67; GL261 = 83.22; 0308 = 561.42.
(JPG)

Figure S5 **Effect of variable delay to radiation on clonogenic survival after graded chronic hypoxia (GCH).** Raw clonogenic data, expressed as the number of surviving colonies, are shown for cells exposed to radiation under continuous normoxia (NOx), or graded chronic hypoxia with reoxygenation and return to hypoxia for 1 hour (1 h), 3 hours (3 h), or 6 hours (6 h). Each data point (solid circle) represents the average of three replicates within a given experiment. Three independent experiments were run for each condition and the average value for the three experiments is shown as a red triangle. Statistical assessments for group differences used the Holm-Sidak test for multiple comparisons. The statistical comparisons performed on the raw data are presented in Figure 5B. Note that the data presentation for average group values in Figure 5B is normalized as a percentage of the average clonogenic survival of the negative control for a given cell type. Normalization of the data in this manner allows for presentation on a common y-axis and facilitates group comparisons. The average clonogenic survival of the negative group for each cell type was: U87 = 91.78; U87-luc = 90.11; GL261 = 86.67; 0308 = 562.89.
(TIFF)

Figure S6 **Effect of variable delay to radiation on clonogenic survival after rapid acute hypoxia (RAH).** Raw clonogenic data, expressed as the number of surviving colonies, are shown for cells exposed to radiation under continuous normoxia (NOx), or rapid acute hypoxia with reoxygenation and return to hypoxia for 1 hour (1 h), 3 hours (3 h), or 6 hours (6 h). Each data point (solid circle) represents the average of three replicates within a given experiment. Three independent experiments were run for each condition and the average value for the three experiments is shown as a red triangle. Statistical assessments for group differences used the Holm-Sidak test for multiple comparisons. The statistical comparisons performed on the raw data are presented in Figure 6B. Note that the data presentation for average group values in Figure 6B is normalized as a percentage of the average clonogenic survival of the negative control for a given cell type. Normalization of the data in this manner allows for presentation on a common y-axis and facilitates group comparisons. The average clonogenic survival of the negative group for each cell type was: U87 = 91.78; U87-luc = 90.11; GL261 = 86.67; 0308 = 562.89.
(JPG)

Figure S7 **Effect of HIF-1α overexpression on clonogenic survival after graded chronic hypoxia (GCH) or rapid acute hypoxia (RAH).** Raw clonogenic data, expressed as the number of surviving colonies, are shown for U87 cells transfected with either an empty vector or HIF-1α expression vector and then exposed to radiation under continuous normoxia (NOx), or GCH or RAH protocols without (–) or with (+) reoxygenation. Each data point (solid circle) represents the average of three replicates within a given experiment. Three independent experiments were run for each condition and the average value for the three experiments is shown as a red triangle. Statistical assessments for group differences used the Holm-Sidak test for multiple comparisons. The statistical comparisons performed on the raw data are presented in Figure 7B. Note that the data presentation for average group values in Figure 7B is normalized as a percentage of the average clonogenic survival of the negative control for a given cell type. Normalization of the data in this manner allows for presentation on a common y-axis and facilitates group comparisons. The average clonogenic survival of the negative group for each cell type was: U87 w/empty vector = 66.33; U87 with HIF-1α expression vector = 57.89.
(TIFF)

Figure S8 **Effect of transient hyperoxia prior to radiation on clonogenic survival.** Raw clonogenic data, expressed as the number of surviving colonies, are shown for cells exposed to radiation under continuous normoxia (NOx), or following transient hyperoxia (HyperOx). Each data point (solid circle) represents the average of three replicates within a given experiment. Three independent experiments were run for each condition and the average value for the three experiments is shown as a red triangle. Statistical comparisons of the two groups for a given cell type utilized Student’s t-test. The statistical comparisons performed on the raw data are presented in Figure 8B. Note that the data presentation for average group values in Figure 8B is normalized as a percentage of the average clonogenic survival of the negative control for a given cell type. Normalization of the data in this manner allows for presentation on a common y-axis and facilitates group comparisons. The average clonogenic survival of the negative group for each cell type was: U87 = 91.78; U87-luc = 90.11; GL261 = 86.67; 0308 = 562.89; normal human astrocytes = 121.89.
(JPG)

Table S1 **Effect of hyperoxic pretreatment on animal survival.** The time courses of survival of nude mice injected with U87-luc cells are shown for four treatment groups: 0 Gy+21% O_2_, 0 Gy+100% O_2_, 8 Gy+21% O_2_, and 8 Gy+100% O_2_ and 8 Gy+100% O_2_. The data are presented as a percentage of the animals surviving on each day post-tumor implant starting on Day 25 and continuing until Day 60. Oxygen and/or radiation treatments were administered on Day 14 post-tumor implant.
(TIFF)

Table S2 **Tumor growth slopes for individual animals.** Slope values for tumor growth were generated from the linear regression plots of IVIS measurements presented in Figure S2. Slope values are shown for each animal in the 8 Gy+21% O_2_ and 8 Gy+100% O_2_ treatment groups.
(JPG)
