# Supplementary figures and images for: Sustained Radiosensitization of Hypoxic Glioma Cells after Oxygen Pretreatment in an Animal Model of Glioblastoma and In Vitro Models of Tumor Hypoxia
Source: PLoS One. 2014 Oct 28;9(10):e111199. doi: 10.1371/journal.pone.0111199 (PMC4211739; doi:10.1371/journal.pone.0111199)

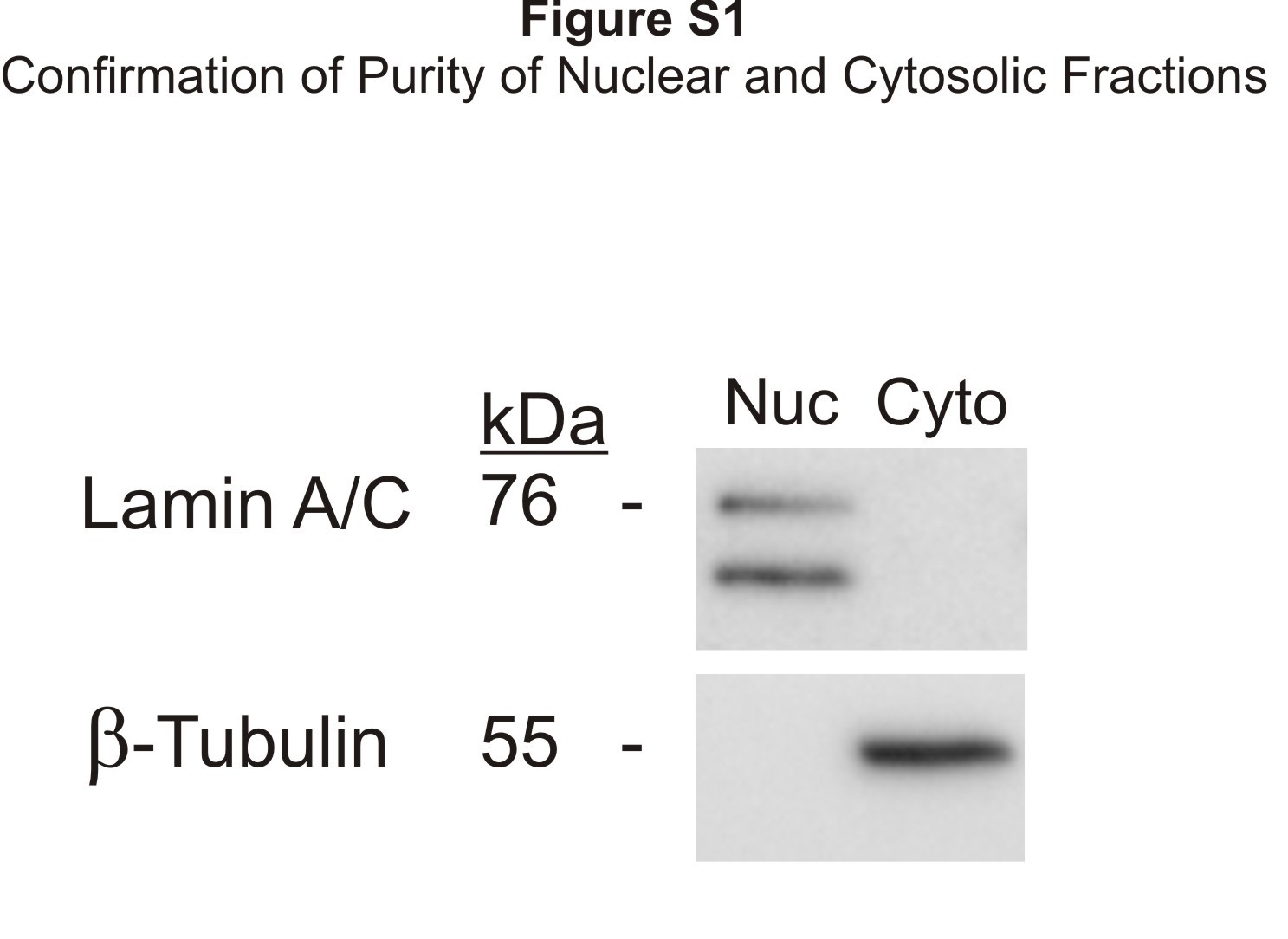

Supplement: Figure S1 — Confirmation of purity of nuclear and cytosolic fractions. In order to demonstrate purity of nuclear and cytosolic fractions generated from whole cell lysates, representative Western blot images are shown. Nuclear (Nuc) and cytosolic (Cyto) fractions from the U87 NOx sample were subjected to SDS-PAGE and Western analysis. Blots were probed using appropriate loading control antibodies for nucleus-specific (lamin A/C) and cytosol-specific (β-tubulin) proteins. Lamin A/C was highly enriched in the nuclear fraction, while β-tubulin was not detected. In contrast, β-tubulin was enriched in the cytosolic fraction, while lamin A/C was not detected. (JPG) [file pone.0111199.s001.jpg]

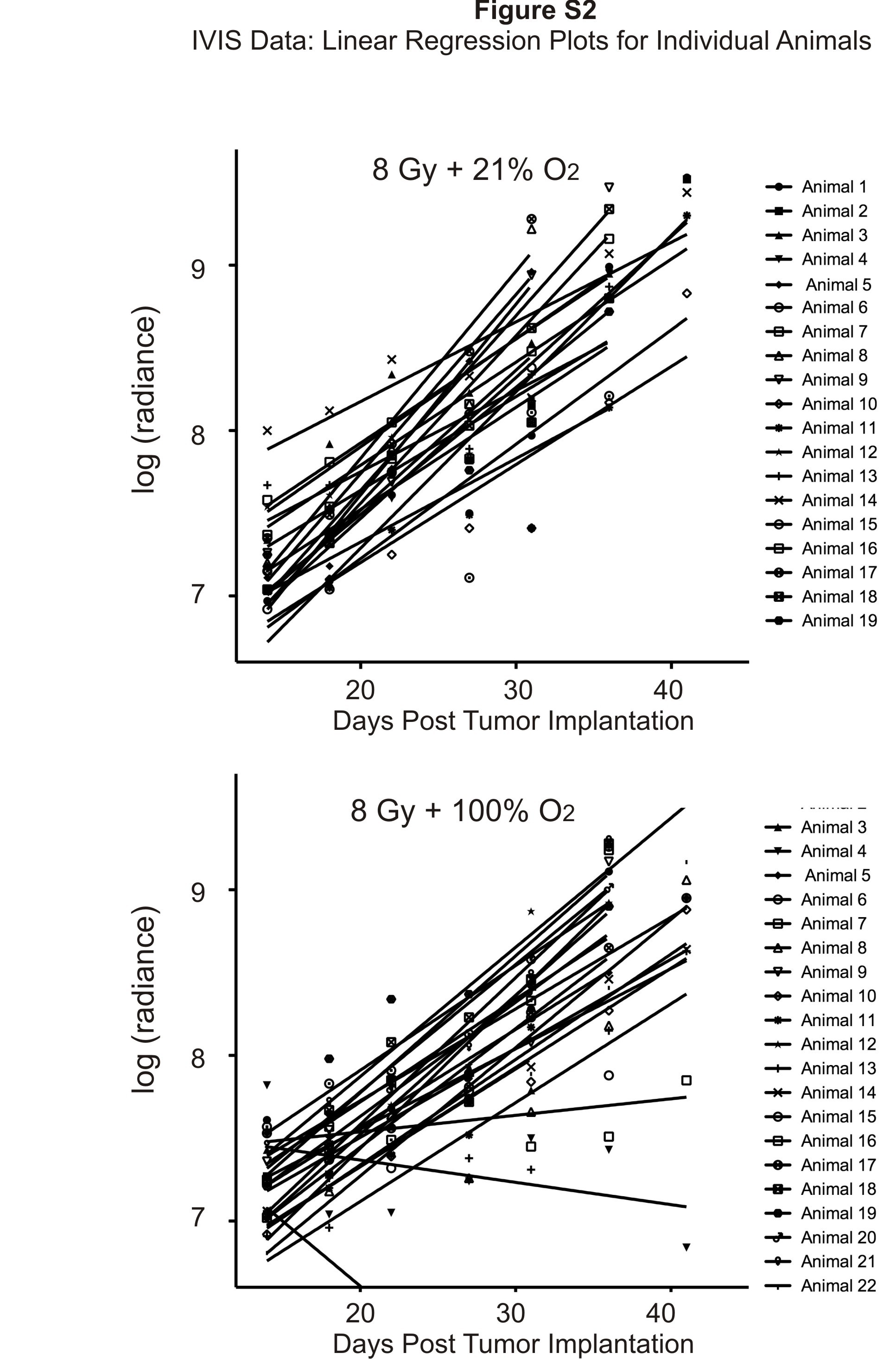

Supplement: Figure S2 — Tumor growth plots: linear regressions for individual animals. Linear regression plots of tumor growth over time are shown for animals in the 8 Gy+21% O2 and 8 Gy+100% O2 treatment groups. Individual data points represent the log of tumor radiance, as assessed by IVIS at each tumor measurement time point. Slope values for tumor growth slope were generated from these linear regression plots and are shown in Table S2. (JPG) [file pone.0111199.s002.jpg]

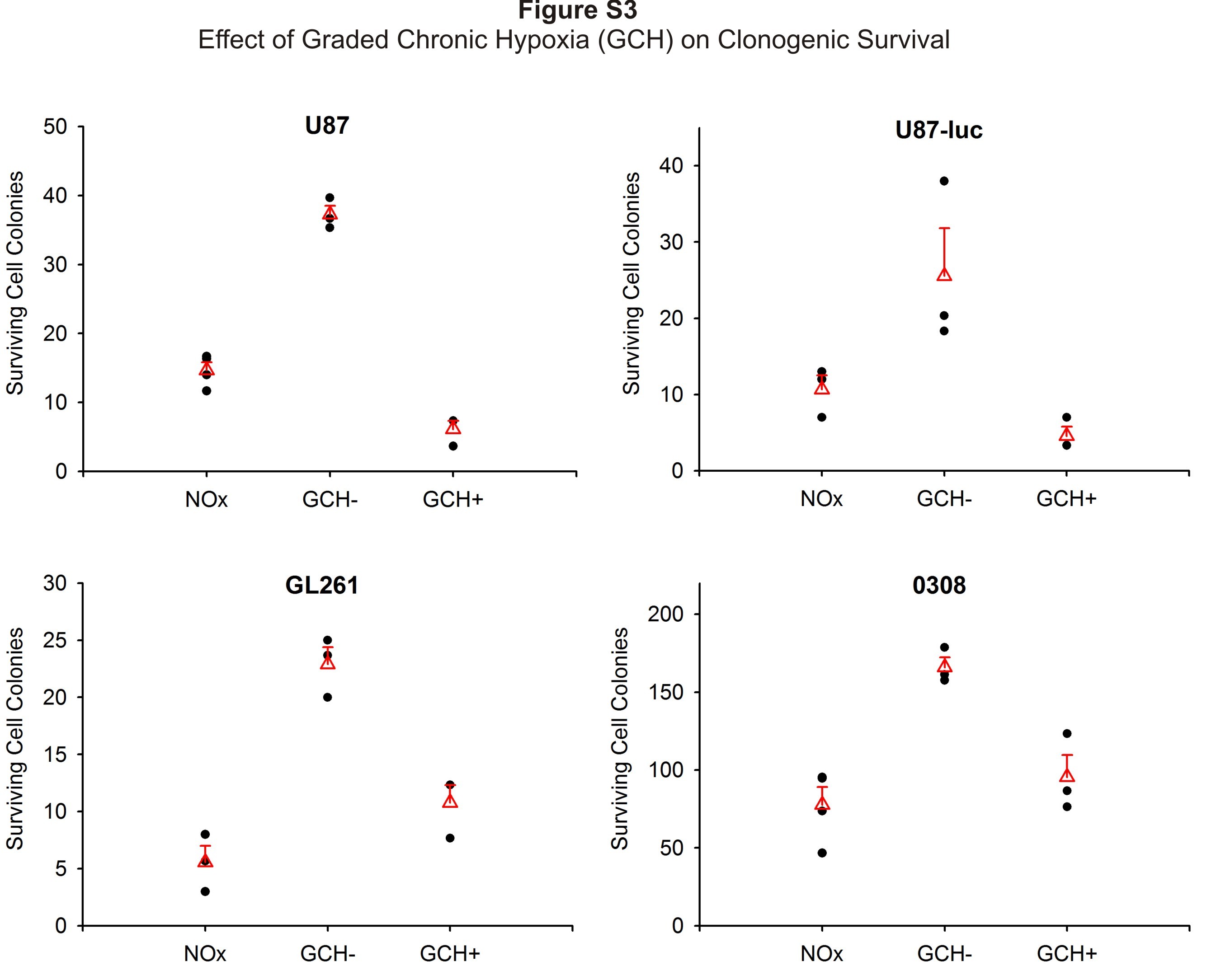

Supplement: Figure S3 — Effect of graded chronic hypoxia (GCH) on clonogenic survival. Raw clonogenic data, expressed as the number of surviving colonies, are shown for cells exposed to radiation under continuous normoxia (NOx), graded chronic hypoxia without reoxygenation (GCH−), or graded chronic hypoxia with reoxygenation (GCH+). Each data point (solid circle) represents the average of three replicates within a given experiment. Three independent experiments were run for each condition and the average value for the three experiments is shown as a red triangle. Statistical assessments for group differences used the Holm-Sidak test for multiple comparisons. The statistical comparisons performed on the raw data are presented in Figure 3B. Note that the data presentation for average group values in Figure 3B is normalized as a percentage of the average clonogenic survival of the negative control for a given cell type. Normalization of the data in this manner allows for presentation on a common y-axis and facilitates group comparisons. The average clonogenic survival of the negative group for each cell type was: U87 = 109.92; U87-luc = 91.67; GL261 = 83.22; 0308 = 561.42. (JPG) [file pone.0111199.s003.jpg]

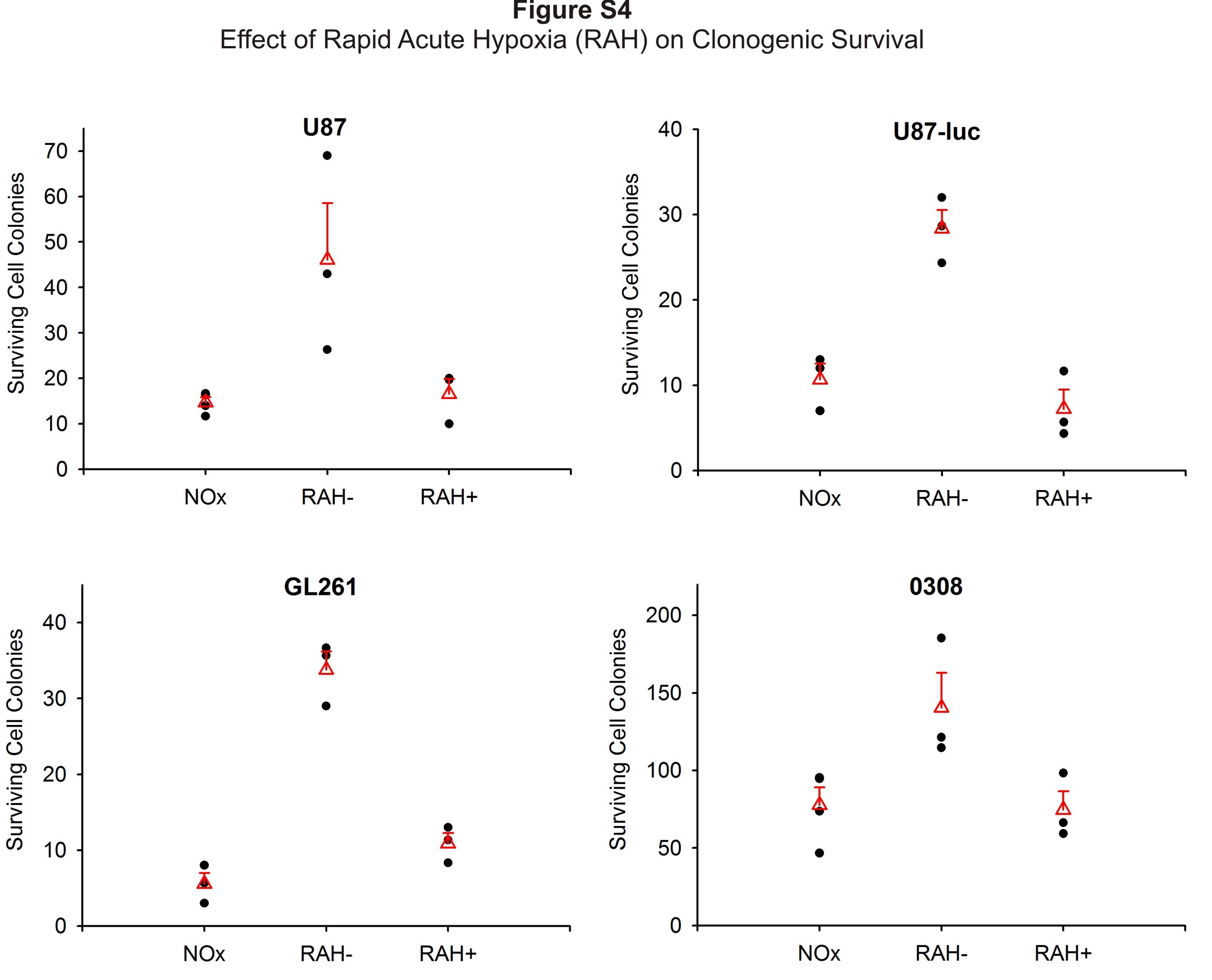

Supplement: Figure S4 — Effect of rapid acute hypoxia (RAH) on clonogenic survival. Raw clonogenic data, expressed as the number of surviving colonies, are shown for cells exposed to radiation under continuous normoxia (NOx), rapid acute hypoxia without reoxygenation (RAH−), or rapid acute hypoxia with reoxygenation (RAH+). Each data point (solid circle) represents the average of three replicates within a given experiment. Three independent experiments were run for each condition and the average value for the three experiments is shown as a red triangle. Statistical assessments for group differences used the Holm-Sidak test for multiple comparisons. The statistical comparisons performed on the raw data are presented in Figure 4B. Note that the data presentation for average group values in Figure 4B is normalized as a percentage of the average clonogenic survival of the negative control for a given cell type. Normalization of the data in this manner allows for presentation on a common y-axis and facilitates group comparisons. The average clonogenic survival of the negative group for each cell type was: U87 = 109.92; U87-luc = 91.67; GL261 = 83.22; 0308 = 561.42. (JPG) [file pone.0111199.s004.jpg]

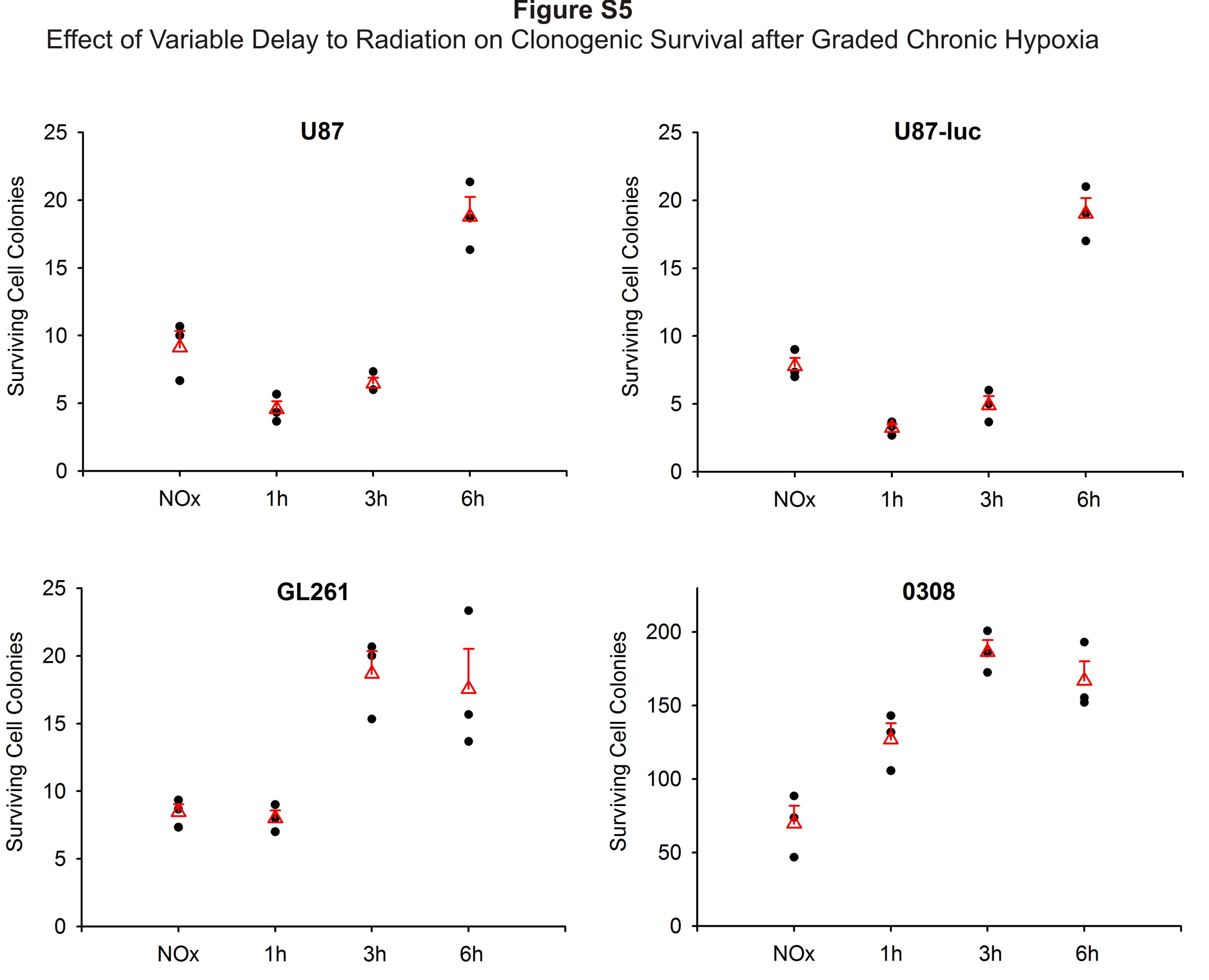

Supplement: Figure S5 — Effect of variable delay to radiation on clonogenic survival after graded chronic hypoxia (GCH). Raw clonogenic data, expressed as the number of surviving colonies, are shown for cells exposed to radiation under continuous normoxia (NOx), or graded chronic hypoxia with reoxygenation and return to hypoxia for 1 hour (1 h), 3 hours (3 h), or 6 hours (6 h). Each data point (solid circle) represents the average of three replicates within a given experiment. Three independent experiments were run for each condition and the average value for the three experiments is shown as a red triangle. Statistical assessments for group differences used the Holm-Sidak test for multiple comparisons. The statistical comparisons performed on the raw data are presented in Figure 5B. Note that the data presentation for average group values in Figure 5B is normalized as a percentage of the average clonogenic survival of the negative control for a given cell type. Normalization of the data in this manner allows for presentation on a common y-axis and facilitates group comparisons. The average clonogenic survival of the negative group for each cell type was: U87 = 91.78; U87-luc = 90.11; GL261 = 86.67; 0308 = 562.89. (TIFF) [file pone.0111199.s005.tiff]

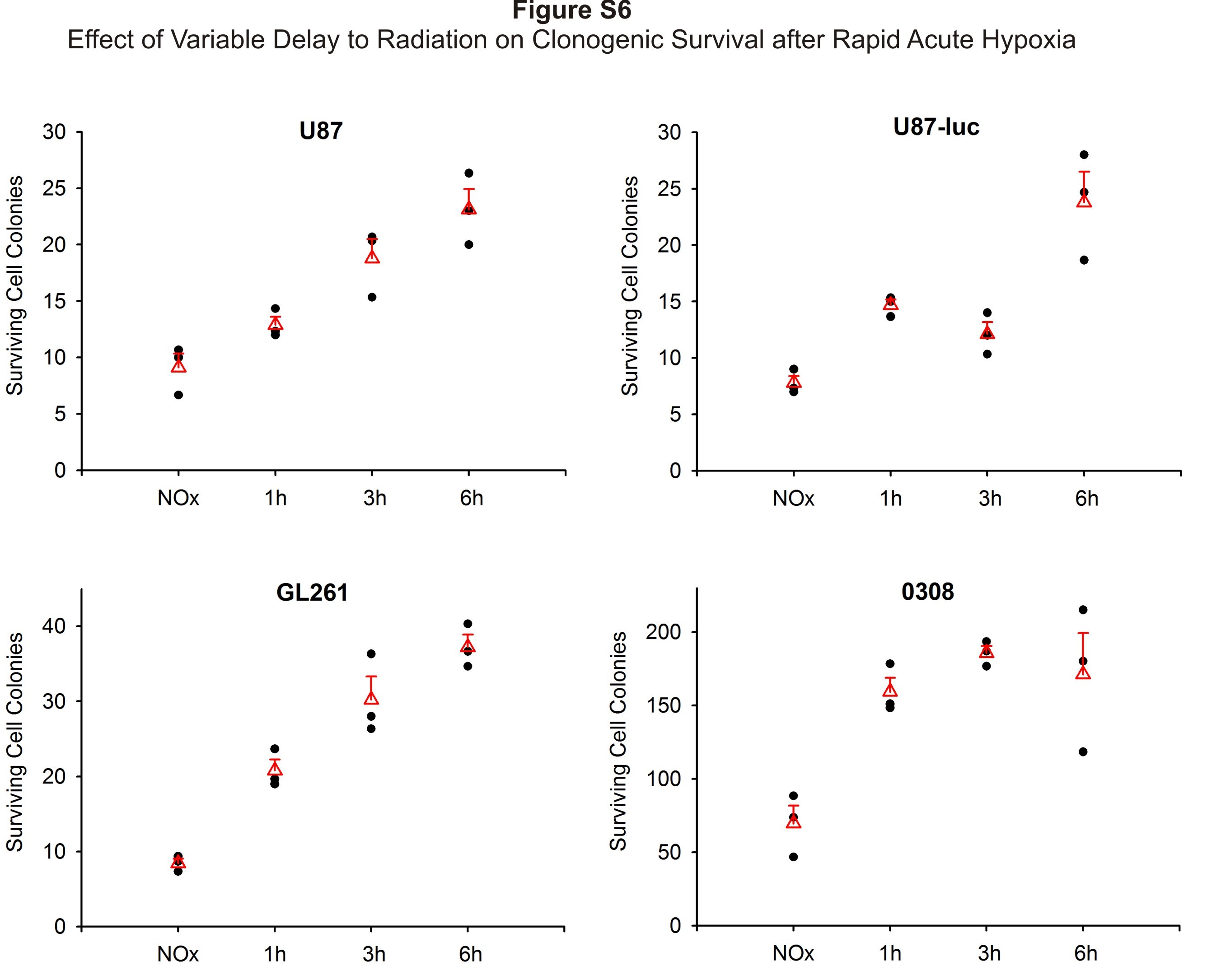

Supplement: Figure S6 — Effect of variable delay to radiation on clonogenic survival after rapid acute hypoxia (RAH). Raw clonogenic data, expressed as the number of surviving colonies, are shown for cells exposed to radiation under continuous normoxia (NOx), or rapid acute hypoxia with reoxygenation and return to hypoxia for 1 hour (1 h), 3 hours (3 h), or 6 hours (6 h). Each data point (solid circle) represents the average of three replicates within a given experiment. Three independent experiments were run for each condition and the average value for the three experiments is shown as a red triangle. Statistical assessments for group differences used the Holm-Sidak test for multiple comparisons. The statistical comparisons performed on the raw data are presented in Figure 6B. Note that the data presentation for average group values in Figure 6B is normalized as a percentage of the average clonogenic survival of the negative control for a given cell type. Normalization of the data in this manner allows for presentation on a common y-axis and facilitates group comparisons. The average clonogenic survival of the negative group for each cell type was: U87 = 91.78; U87-luc = 90.11; GL261 = 86.67; 0308 = 562.89. (JPG) [file pone.0111199.s006.jpg]

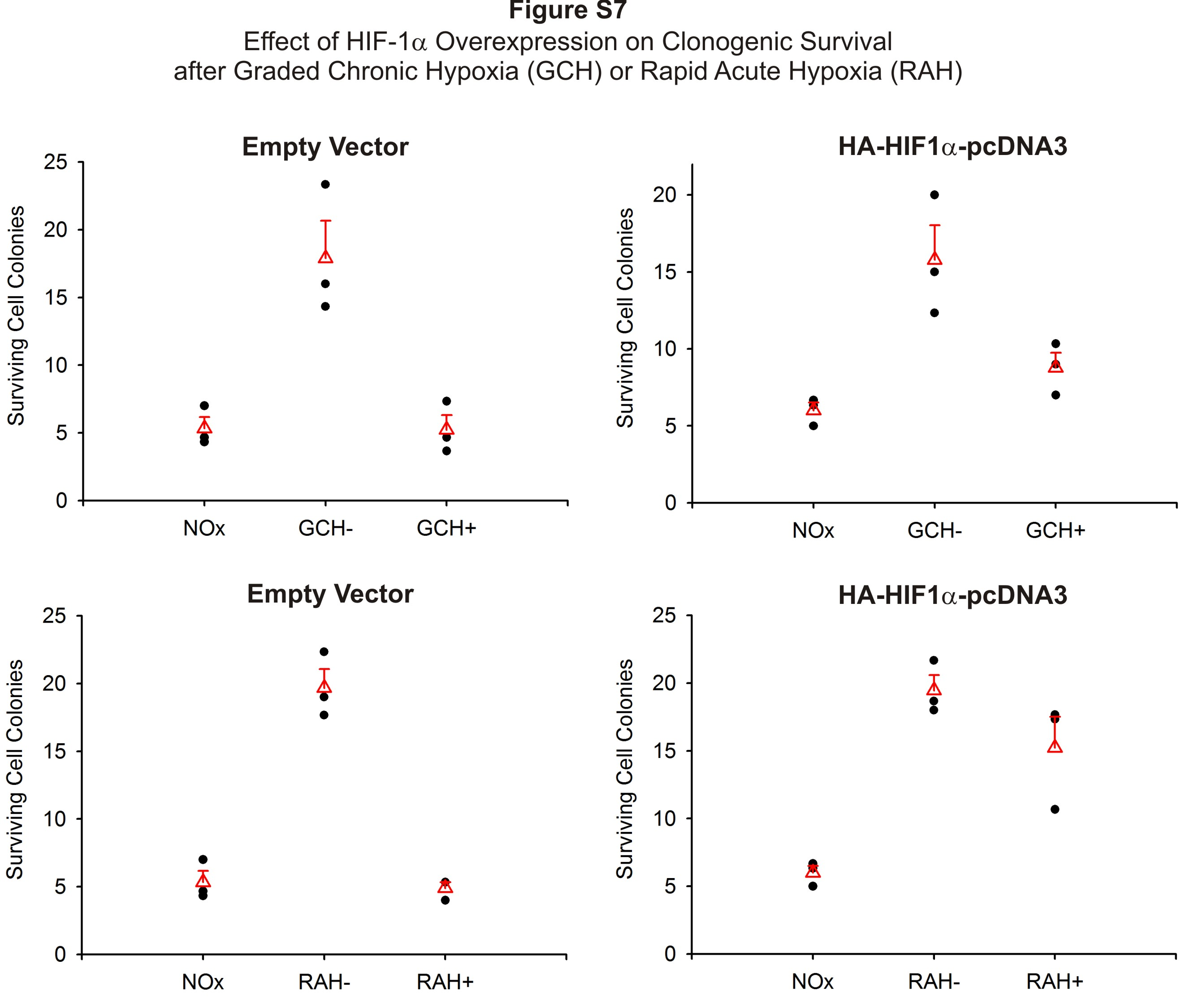

Supplement: Figure S7 — Effect of HIF-1α overexpression on clonogenic survival after graded chronic hypoxia (GCH) or rapid acute hypoxia (RAH). Raw clonogenic data, expressed as the number of surviving colonies, are shown for U87 cells transfected with either an empty vector or HIF-1α expression vector and then exposed to radiation under continuous normoxia (NOx), or GCH or RAH protocols without (–) or with (+) reoxygenation. Each data point (solid circle) represents the average of three replicates within a given experiment. Three independent experiments were run for each condition and the average value for the three experiments is shown as a red triangle. Statistical assessments for group differences used the Holm-Sidak test for multiple comparisons. The statistical comparisons performed on the raw data are presented in Figure 7B. Note that the data presentation for average group values in Figure 7B is normalized as a percentage of the average clonogenic survival of the negative control for a given cell type. Normalization of the data in this manner allows for presentation on a common y-axis and facilitates group comparisons. The average clonogenic survival of the negative group for each cell type was: U87 w/empty vector = 66.33; U87 with HIF-1α expression vector = 57.89. (TIFF) [file pone.0111199.s007.tiff]

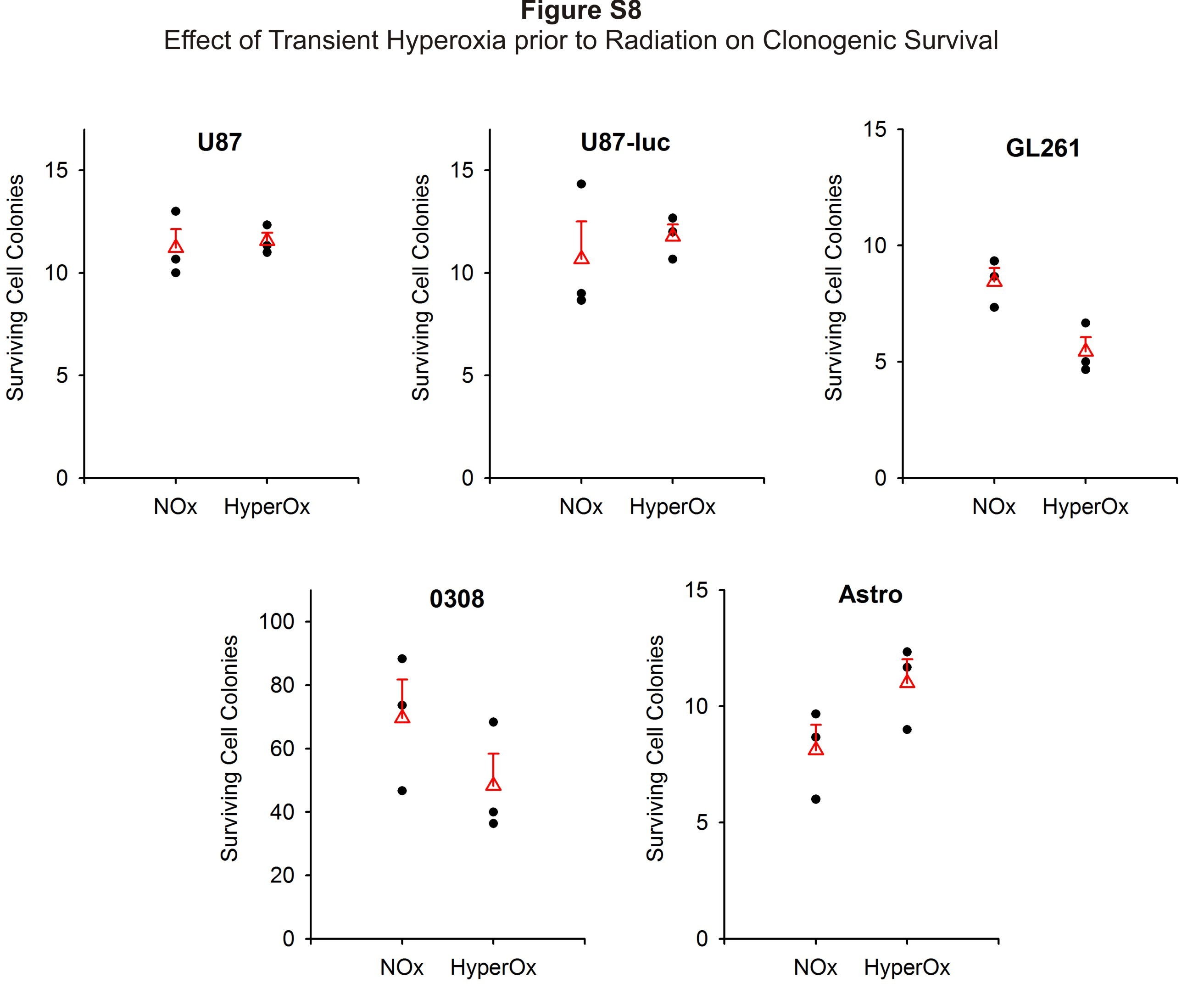

Supplement: Figure S8 — Effect of transient hyperoxia prior to radiation on clonogenic survival. Raw clonogenic data, expressed as the number of surviving colonies, are shown for cells exposed to radiation under continuous normoxia (NOx), or following transient hyperoxia (HyperOx). Each data point (solid circle) represents the average of three replicates within a given experiment. Three independent experiments were run for each condition and the average value for the three experiments is shown as a red triangle. Statistical comparisons of the two groups for a given cell type utilized Student’s t-test. The statistical comparisons performed on the raw data are presented in Figure 8B. Note that the data presentation for average group values in Figure 8B is normalized as a percentage of the average clonogenic survival of the negative control for a given cell type. Normalization of the data in this manner allows for presentation on a common y-axis and facilitates group comparisons. The average clonogenic survival of the negative group for each cell type was: U87 = 91.78; U87-luc = 90.11; GL261 = 86.67; 0308 = 562.89; normal human astrocytes = 121.89. (JPG) [file pone.0111199.s008.jpg]

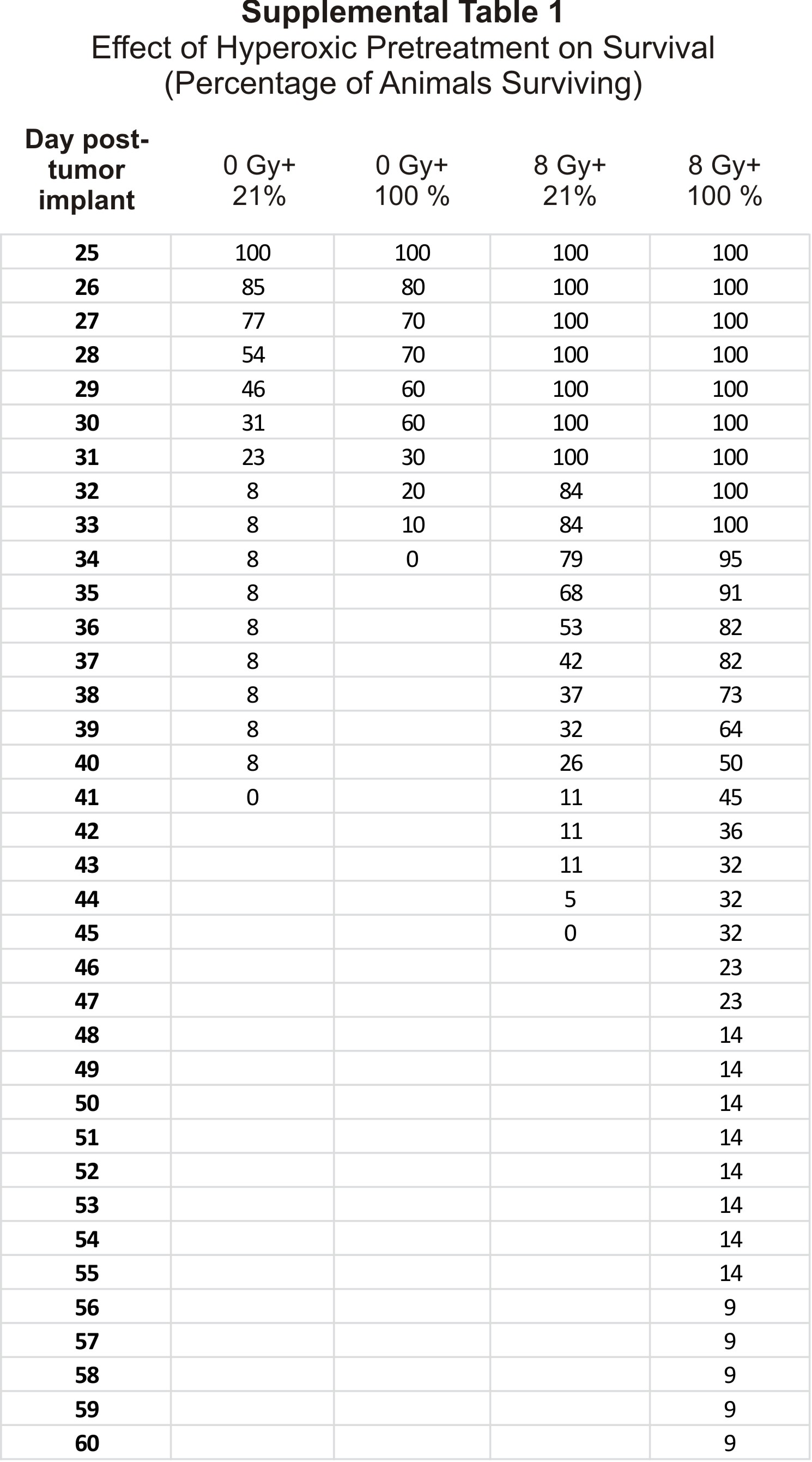

Supplement: Table S1 — Effect of hyperoxic pretreatment on animal survival. The time courses of survival of nude mice injected with U87-luc cells are shown for four treatment groups: 0 Gy+21% O2, 0 Gy+100% O2, 8 Gy+21% O2, and 8 Gy+100% O2 and 8 Gy+100% O2. The data are presented as a percentage of the animals surviving on each day post-tumor implant starting on Day 25 and continuing until Day 60. Oxygen and/or radiation treatments were administered on Day 14 post-tumor implant. (TIFF) [file pone.0111199.s009.tiff]

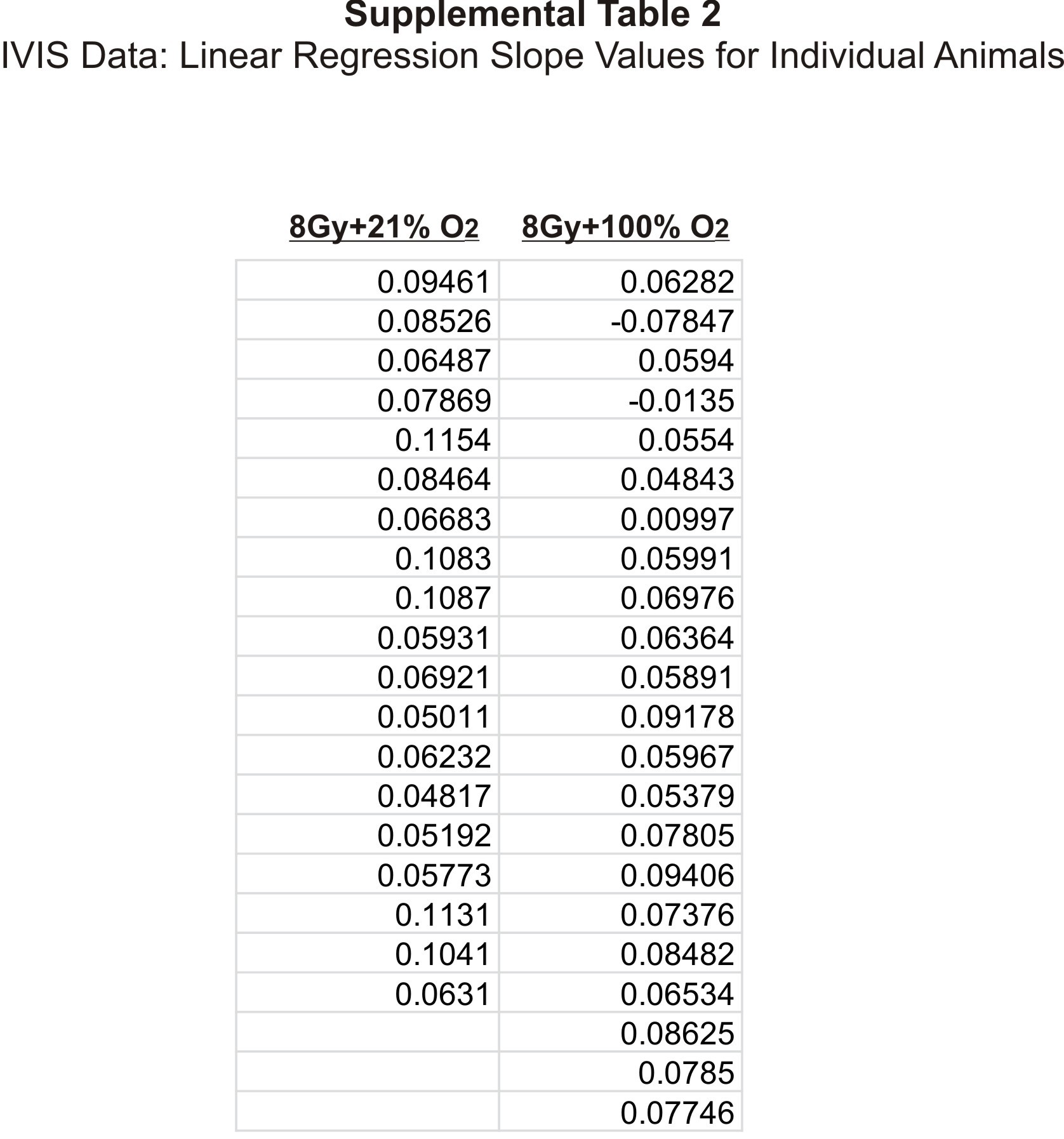

Supplement: Table S2 — Tumor growth slopes for individual animals. Slope values for tumor growth were generated from the linear regression plots of IVIS measurements presented in Figure S2. Slope values are shown for each animal in the 8 Gy+21% O2 and 8 Gy+100% O2 treatment groups. (JPG) [file pone.0111199.s010.jpg]
